# Relational Network for Knowledge Discovery through Heterogeneous Biomedical and Clinical Features

**DOI:** 10.1038/srep29915

**Published:** 2016-07-18

**Authors:** Huaidong Chen, Wei Chen, Chenglin Liu, Le Zhang, Jing Su, Xiaobo Zhou

**Affiliations:** 1 School of Computer and Information Science, Southwest University, Chongqing 400715, China; 2 Center for Bioinformatics & Systems Biology, Division of Radiological Sciences, Wake Forest School of Medicine, Winston-Salem, NC, 27127, USA

## Abstract

Biomedical big data, as a whole, covers numerous features, while each dataset specifically delineates part of them. “Full feature spectrum” knowledge discovery across heterogeneous data sources remains a major challenge. We developed a method called bootstrapping for unified feature association measurement (BUFAM) for pairwise association analysis, and relational dependency network (RDN) modeling for global module detection on features across breast cancer cohorts. Discovered knowledge was cross-validated using data from Wake Forest Baptist Medical Center’s electronic medical records and annotated with BioCarta signaling signatures. The clinical potential of the discovered modules was exhibited by stratifying patients for drug responses. A series of discovered associations provided new insights into breast cancer, such as the effects of patient’s cultural background on preferences for surgical procedure. We also discovered two groups of highly associated features, the HER2 and the ER modules, each of which described how phenotypes were associated with molecular signatures, diagnostic features, and clinical decisions. The discovered “ER module”, which was dominated by cancer immunity, was used as an example for patient stratification and prediction of drug responses to tamoxifen and chemotherapy. BUFAM-derived RDN modeling demonstrated unique ability to discover clinically meaningful and actionable knowledge across highly heterogeneous biomedical big data sets.

The explosive expansion of biomedical big data^1^,^2^,^3^ provides enormous opportunities for translational research such as precision medicine^4^ if the new challenges of data mining and knowledge discovery can be addressed. Public data reservoirs such as the Gene Expression Omnibus (GEO), the database of Genotypes and Phenotypes (dbGaP), etc., have hosted various datasets generated from diverse sources, from publication-associated data deposition to collective efforts of systematic data generation such as The Cancer Genome Atlas (TCGA). Such modern biomedical big data^5^,^6^,^7^,^8^ demonstrate a pair of controversial characteristics: the “global abundance” *versus* the “local deficiency” of features across datasets, which raise unique challenges in data mining that have not yet been well studied.

Global abundance of biomedical features is one of the most appealing characteristics of biomedical big data. As a whole, biomedical big data provide multi-scale and diverse descriptions of biological or pathological events at different “throughput levels” across a range of experimental platforms and clinical systems. For example, in the TCGA dataset, breast cancer has been profiled at molecular, cellular, tissue, patient, and population levels in terms of biological traits as well as drug and treatment responses in short-term (pathological complete response) and long-term (distant metastasis-free survival) contexts. Types of data range from high-throughput omics assays to conventional demographic and diagnostic features. Besides the research and trial data collected from specific cohorts^9^,^10^,^11^,^12^, electronic medical records (EMRs), which are free of study-specific biases, provide valuable and independent information on patients, diseases, treatments, and outcomes^13^,^14^,^15^. Thus, biomedical big data as a whole has extremely rich features enabling researchers, for the first time, to investigate a disease across the full spectrum of features in a way that any individual dataset cannot.

However, individual biomedical big datasets demonstrate “local deficiency” of features that, if not addressed, will cause a *“drinking seawater” paradox* and limit full-spectrum analysis of rich features. For features available in a pan-dataset analysis, each dataset only covers a small portion of them. Such intrinsic “local deficiency” of features in each dataset and heterogeneity of feature availability across biomedical big datasets raises unique challenges and demands novel approaches for data mining. This limitation causes a *“drinking seawater” paradox*: the more the seawater is consumed to ease thirst, the more water in the body will be lost, and the worse the thirst will be; similarly, the more that biomedical big datasets with heterogeneous features are incorporated, the fewer features and samples are qualified for analysis, and the less knowledge can be discovered. This paradox stems from the heterogeneous coverage of features across datasets. Imagine that a virtual “data table” for knowledge discovery was established by incorporating multiple biomedical big datasets for breast cancer (Figure S1, features in rows and datasets in columns). This virtual table could have millions of features (rows), but each dataset (column) would only cover a small portion of them (grids labeled in orange), leaving most part of the table empty (grids labeled in grey). For example, the correlations among features A, B, and E can be analyzed with dataset 1; however, after data integration, the values of these three features are missing for most samples, making any global association analyses of these features challenging. Generally speaking, as more datasets are combined, the coverage ratio of a feature over all samples tends to decrease.

Missing value imputation approaches such as k-nearest-neighbor^16^,^17^ and random forest^18^ algorithms, which are widely used in biomedical data preprocessing, are not suitable to address such a paradox. Such approaches assume “nearly complete” (only a modest amount of missing values) data tables *randomly* distributed across features. Complete data harvest is designed, and missing data are due to random causes such as poor data quality (for example, stains and poor signals in raw microarray data) or human errors (e.g., occasionally missed or conflicting records in EMRs). However, for “structurally missed features”, as demonstrated in Figure S1, data for some features are totally missing in some datasets. In this situation, imputation approaches will significantly deteriorate the quality of the data^19^.

Another strategy is to combine local knowledge discovery in individual datasets and global summaries of the results crossing datasets^20^, such as meta-analysis approaches^21^,^22^,^23^. However, when the samples that support the evaluation of an association between two features are distributed into multiple datasets, statistical power may be significantly impaired beyond the compensation strategies used in the summary algorithms. Intrinsic variations among datasets are also a challenge, because statistical measures are often not comparable across datasets, even for the same type of data^23^.

Pairwise association analysis^24^,^25^, which utilizes all available data shared by a pair of features, also provides solutions to address local feature deficiency. However, currently, most pairwise approaches are focused on homogeneous associations within the same dataset. A statistical measure to fairly compare heterogeneous associations across datasets would increase the utility of pairwise association analyses. Meanwhile, global summarization approaches are particularly important for understanding and further extending the results of pairwise association analysis, but these are largely underdeveloped.

To address this “drinking seawater paradox”, we developed the relational dependency network (RDN) of biomedical features based on bootstrapping for unified feature association measurement (BUFAM). BUFAM provided a *uniform* metric to enable unbiased analysis of pairwise feature associations *across* datasets, whereas RDN allowed global analysis and summarization of the knowledge discovered by BUFAM. Thus, the BUFAM-derived RDN model provides a general solution for the feature deficiency problem during the integration of biomedical big data sets of heterogeneous features. Such strategy enables efficient and effective use of heterogeneous biomedical big data for understanding diseases and optimizing clinical decisions.

The design of the BUFAM-derived RDN approach is illustrated in Figure S1. Multiple datasets are used to infer the relations among features 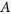, B, C, D, and E of different data types, whose true relations are demonstrated as a relational network (Figure S1, lower left corner). The association between each pair of features is examined using samples from *all* datasets that share these two features. Once all associations that are directly testable are profiled, an RDN of discovered associations can be established. Network-based analysis tools can be applied to this RDN to generate a global understanding of the pairwise discovered knowledge, and to further identify associated features that do not share enough data to test direct associations. Feature heterogeneity^2^,^26^,^27^ is the dominating challenge to be addressed in this work.

As illustrated in Figure S3 (Supplement), biomedical big data sets used in this study showed disparate features in terms of: 1) feature types, which included demographic features, diagnostic results, and molecular profiling; 2) data types, which covered numeric, binary, and categorical (nominal and ordinal) types; and 3) local deficiency among datasets (the gray areas in Figure S3). Measuring the associations among different variable types and accurately ranking them to highlight significant ones were the major challenges during this study. Formalized definitions of the question, the goal, and the challenges are provided in Supplement Section 1 (Formalization of the heterogeneous association problem).

## Results and Discussions

### Knowledge discovery by BUFAM

**Figure 1:**
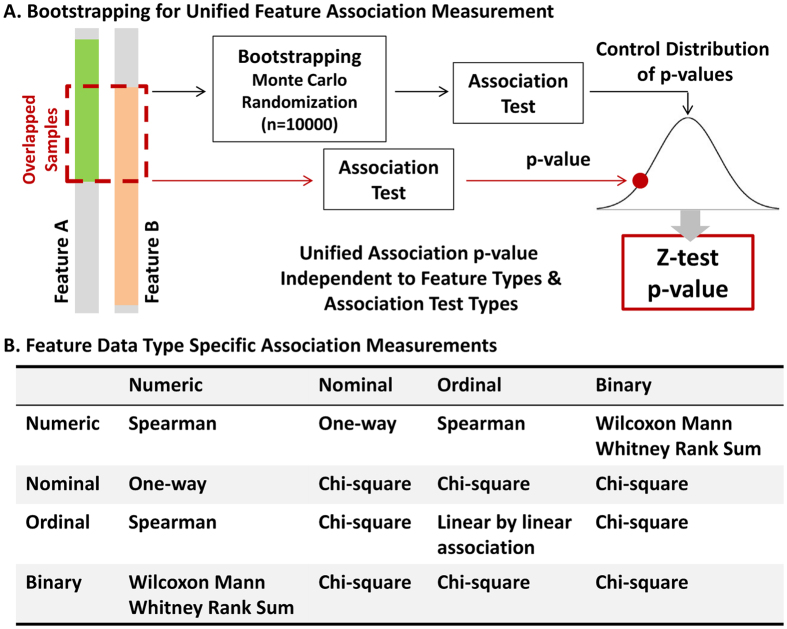
Bootstrapping for unified feature association measurement (BUFAM). (**A**) Flowchart of the BUFAM algorithm. (**B**) Statistical measurements for specific combinations of feature data types. Mathematical details are in the Supplement.

**Figure 2:**
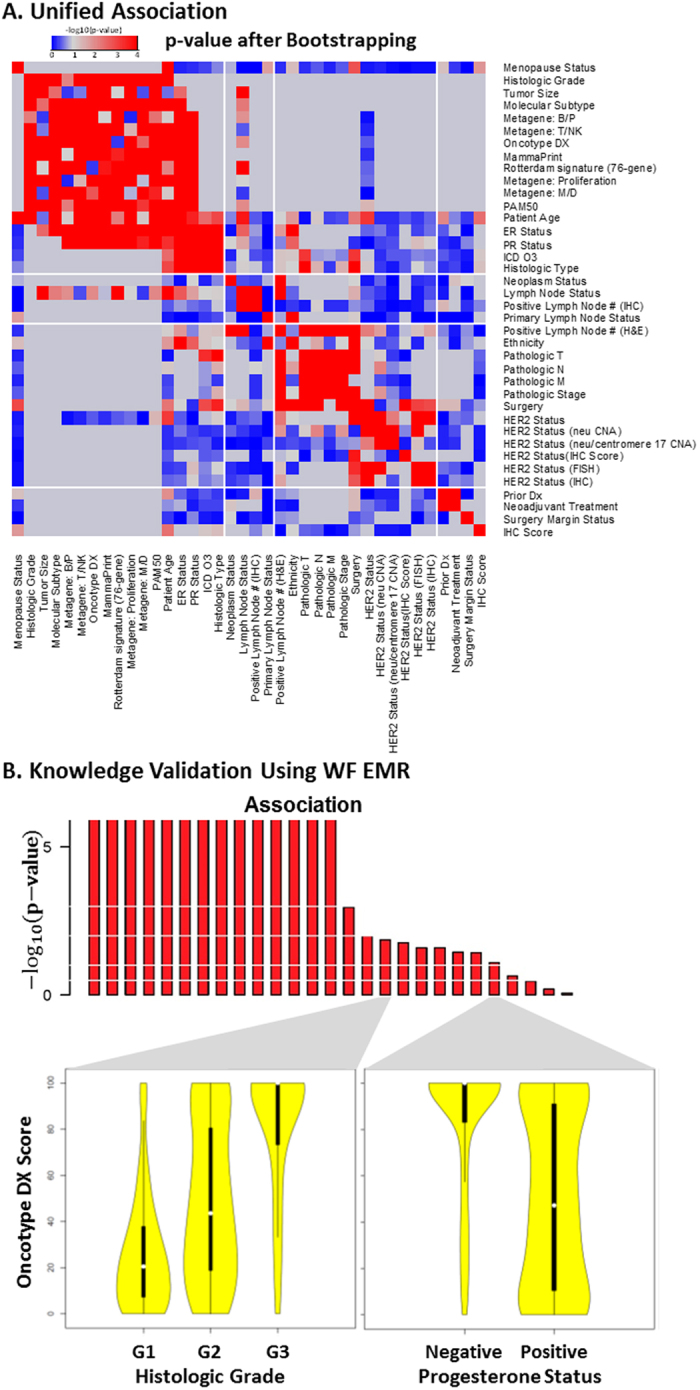
BUFAM feature association discovery. (**A**) Overview of the measures of pairwise feature associations presented in 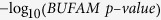. Gray regions represent non-testable associations. **(B)** Validation of discovered associations using the EMR of Wake Forest Baptist Medical Center (WakeOne) presented in 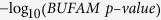. Associations between Oncotype DX score and histologic grade, and between Oncotype DX score and progesterone status, are shown as examples.

The unified relationships among heterogeneous biomedical features, evaluated by BUFAM p-values (Fig. 1A BUFAM approach, and Fig. 1B BUFAM associations, Data and Methods section), are summarized as a heat map in Fig. 2(A) and Figure S1. Strong associations among variables, indicated by smaller p-values (the red regions), were sparse and locally condensed, comparing the green regions (large p-values) with the rest. For example, features in upper left corner with significant p-values were highly related to each other. Among all 27 testable associations discovered by BUFAM, 22 were validated using electronic medical records from Wake Forest Baptist Health (WFU EMR) (Fig. 2B). The Jaccard similarity coefficient (defined by Equation 1in Section 1, Supplement Document) for the shared features of discovery (TCGA/MDACC/WFUCCC) and the validation datasets (WFU EMR) was 0.81.

Our association results were consistent with current knowledge about breast cancer. For example, estrogen receptor (ER) status, an important breast phenotype widely used for patient subtyping and treatment design, was associated with patients’ ethnicity (Fig. 2A). The ER-negative phenotype was more prevalent among African American patients than others in the WFU EMR (Figure S4), consistent with other population studies^28^,^29^.

We also observed an ethnicity-specific preference regarding choices for surgery (Figure S5, p-value < = 0.00023, χ^2^ test), which was not reported before. Asian patients were less likely to choose lumpectomy (7.7%) compared with White (27.4%) and African (32.5%) American patients. However, about 50% of Hispanic patients chose lumpectomy. Hispanic and Asian patients were also less likely to choose either simple or modified radical mastectomy (25% and 28%, respectively), compared to African American (52.5%) and White (55.8%) patients.

The WFU EMR data provided an independent cross-validation for knowledge discovery using public datasets. The patient cohort was local and free from any recruitment bias, since data collection was not research-oriented. The high ratio of validated knowledge suggested that the discovered knowledge from heterogeneous public sources was reliable and remained valid compared to clinical cases.

A major concern in comparing data is the merging of patient data from various sources without specialized data harmonization. On one hand, this strategy would neutralize the population difference among cohorts due to different patient recruitment standards and original research purpose of each project. Thus, as more cohorts are incorporated, fewer cohort-specific patient features would affect the results, and the more representative the discovered associations would be. On the other hand, cohort-specific data collection and processing bias might be introduced and thus cause false-positive findings. However, our EMR validation implied that such a merging strategy would not introduce significant population-related false discovery.

Oncotype DX Score was used as a pivotal feature for evaluating: 1) if batch effects overwhelmed true associations and 2) if the discovered associations were consistent with literature. Originally identified^30^,^31^ using microarray-based gene expression data for 21 genes and later proven reliable compared to microarray data of other cohorts^9^, the Oncotype DX Score had been commercialized and standardized as a real-time RT-PCR (reverse transcriptase-polymerase chain reaction) based clinical assay^32^ and been approved for use in patient diagnoses and treatments. As one of the first FDA-approved molecular signatures for personalized treatments, Oncotype DX Score had been studied by many research groups in various clinical trials. This literature provides relatively reliable information for the purposes of validation, despite inconsistent reports of the performance of Oncotype DX Score.

The 21-gene signature^9^ of the research datasets (TCGA, WFUCCC, and MDACC) were calculated from microarray data, while the Oncotype DX Score data in the WFU EMR were acquired using a PCR-based commercial toolkit. Strong batch effects were expected due to differences in sample preparation, expression assays, data normalization, and score calculation between the microarray-based data and the PCR-based Oncotype DX Score. We theorized that if associations from Oncotype DX Score data were consistent with those from the EMR data, we could demonstrate whether the BUFAM approach was robust to batch effects.

The discovered and validated associations were not only fully consistent, but also consistent with published results. For example, the strong positive association between the Oncotype DX Score with higher histologic grades (Fig. 2B) is consistent with the multiple linear regression analysis by Flanagan and colleagues^33^. We also discovered and validated the finding that patients with progesterone receptor (PR)-positive breast cancer often had lower Oncotype DX Scores (Fig. 2B), consistent with the design of this tool^30^. Therefore, the validated associations related to Oncotype DX suggested that it the BUFAM algorithm was robust to batch effects and produced reliable discoveries.

BUFAM provides a flexible metric platform for customized and scalable data mining. The default statistical tests (Fig. 1B), bootstrapping algorithm, and the Z-test can be replaced to incorporate prior knowledge and improve sensitivity. The pairwise nature of the algorithm makes it suitable for large-scale parallelism using high-performance computation clusters or cloud computation, which is important for mining big data.

### Comparison with KNN imputation

**Figure 3:**
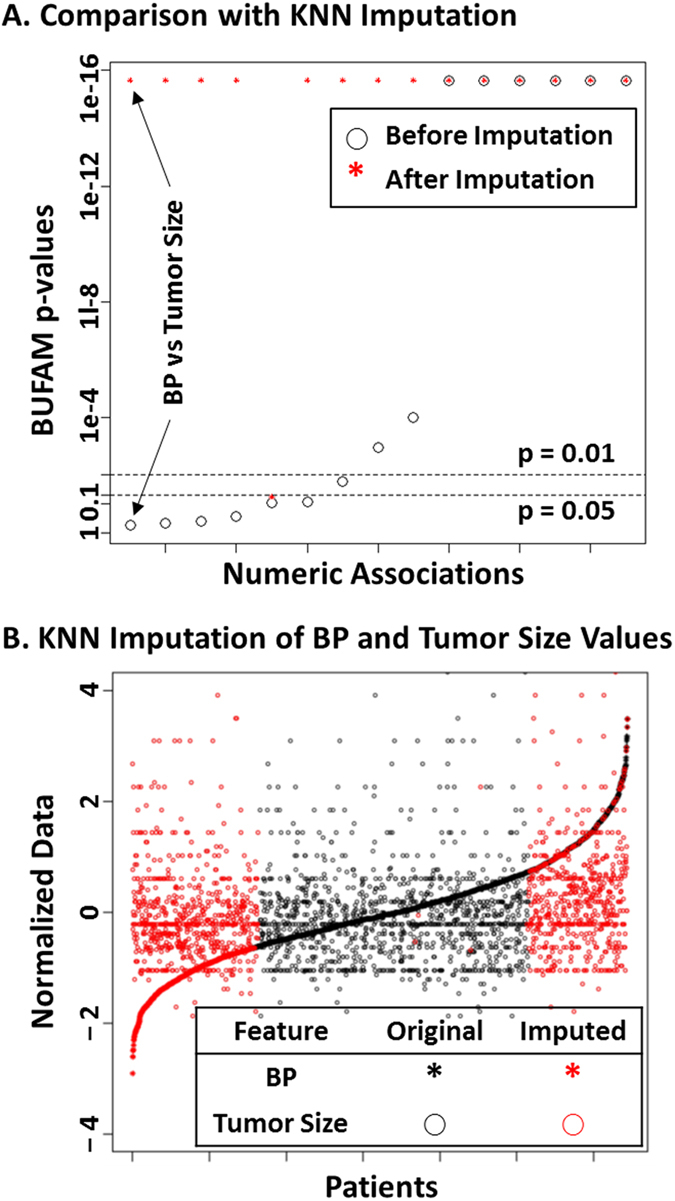
Comparison with KNN algorithm. (**A**) Missing values of 6 numeric features were imputed by the KNN algorithm. These were compared with p-values generated by BUFAM for the corresponding 15 pairwise associations before (black circles) and after (red stars) imputation. Associations were sorted according to p-values generated by BUFAM before imputation. (**B**) Comparisons of normalized original (black) and imputed (red) values of the BP metagene (stars) and tumor size (circles). Patients were sorted according to normalized BP metagene values.

After performing data imputation for numeric features using the KNN algorithm, most pairs of features that demonstrated associations of various strengths before imputation were no longer distinguishable. Among 10 numeric-to-numeric associations, 9 showed apparent strong associations (Fig. 3A). Figure 3B illustrated the impact of KNN imputation on features. Before imputation, the features “Metagene: B/P” and “Tumor Size” exhibited no noticeable relations (black points), consistent with the weak statistical significance (BUFAM p-value) in Fig. 3A (association of BP vs Tumor Size). The imputed data (red points) brought in strong artificial patterns in both BP metagene and tumor size, which explained the strong and false post-imputation association we observed.

Using the KNN algorithm for imputation of numeric data has been one of the most reliable approaches for processing missing data. It has shown robustness against distribution, percentage^34^,^35^, and sources^17^ of missing values. However, the current comparison showed that for *structurally* missed features resulting from differences of feature coverage among datasets, in 80% of cases, current data imputation approaches introduced artifacts that overwhelmed the original data patterns.

Features are not always imputable. An essential assumption for KNN imputation is that features have a strong enough association that the similarity defined by any feature is likely to be consistent. Under this assumption, if a sample is missing a feature, a set of samples with similarity to this sample in all other features (i.e., the “nearest neighbors”) can be used to estimate the missed feature of the first sample. Although this assumption works well for microarray data^17^,^34^,^35^, it has limitations for other applications. Our results showed that strong feature associations (Fig. 2A) were not as prevalent, and therefore some features may not be imputable at all. BUFAM, which does not rely on complete data and imputation, is a more general approach and thus suitable for a wider range of applications.

### Comparison with meta-analysis

**Figure 4:**
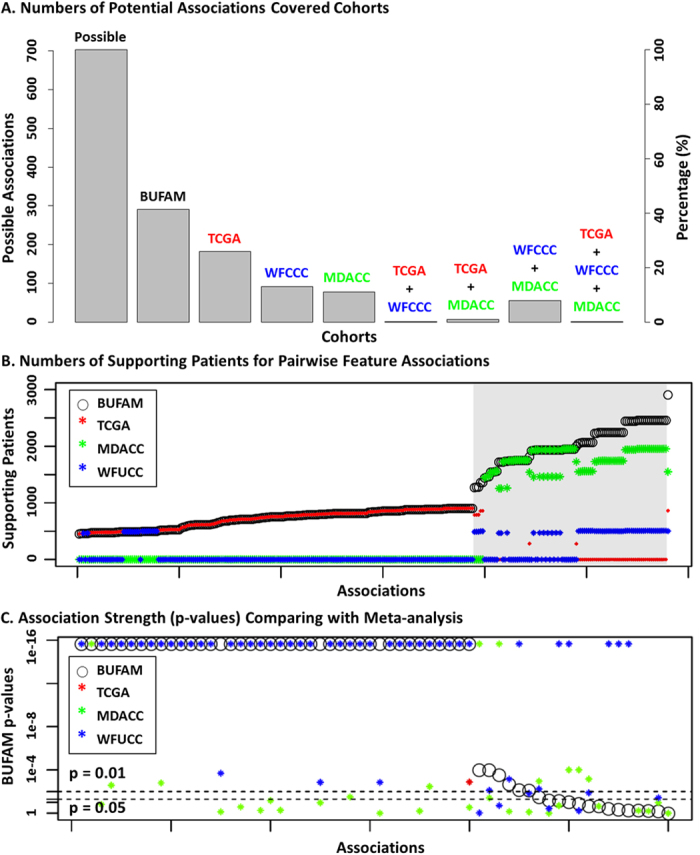
Comparison with meta-analysis. (**A**) Comparison of feature pairs for BUFAM and cohort-based analyses. (**B**) Numbers of supporting patients for each pairwise association found with BUFAM compared to meta-analysis. (**C**) Comparison of association results of BUFAM and meta-analysis were compared. The two horizontal dashed lines marked the p-values of 0.01 and 0.05, respectively. Black: BUFAM; Blue: WFCCC; Green: MDACC; Red: TCGA.

We compared the results of our pan-dataset association analyses with meta-analyses in terms of feature coverage, patient coverage, and association results (Fig. 4). The unified coverage of possible associations using BUFAM was significantly higher than the meta-analysis (Fig. 4A). For each association, the number of the supporting patients (Fig. 4B) was equal to meta-analysis (white region) if only one cohort was available, or significantly higher (gray region) if patients from multiple cohorts share the required feature pairs. In terms of p-values, BUFAM and meta-analyses demonstrated consistent findings (Fig. 4C).

Compared with meta-analyses, the greatest strength of the BUFAM approach was the unified and robust measurement of feature associations across cohorts. Since all associations were assessed using the same strategy, the measurements were less sensitive to the cohorts used. Meanwhile, since more patients could be used for knowledge discovery (Fig. 4B), BUFAM fully utilized the available data and benefited from the increased statistical power. For example, three associations (“Patient Age vs. Metagene: T/NK”, “Patient Age vs. Metagene: Proliferation”, and “MammaPrint vs. Metagene: M/D”, marked by black arrows in Fig. 4C) showed strong associations (black circles, 
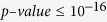
) in BUFAM analysis using the WFCCC and MADCC cohorts (2,462 patients; for availability of the corresponding features, see Figure S3). In meta-analyses, these associations were only detectable in the larger WFCCC cohort (blue stars in Fig. 4C; n = 1,954 patients) with much less statistical significance (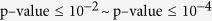) but not in the smaller MDACC cohort (green stars; n = 508 patients). Another specific case, the association between Metagene:Proliferation and patient age, was thoroughly analyzed and externally verified using the TCGA RNA-seq dataset (Supplement Section 7).

Cohort-specific biases, especially the sampling bias of patients, often challenge meta-analysis. BUFAM provides a fair incorporation to minimize such biases. Cohort-specific bias with respect to some features, however, may introduce artifacts and reduce statistical sensitivity of the BUFAM approach. Careful data cleaning and normalization are thus crucial for the sensitivity of BUFAM.

### RDN-based biomedical feature analysis

**Figure 5:**
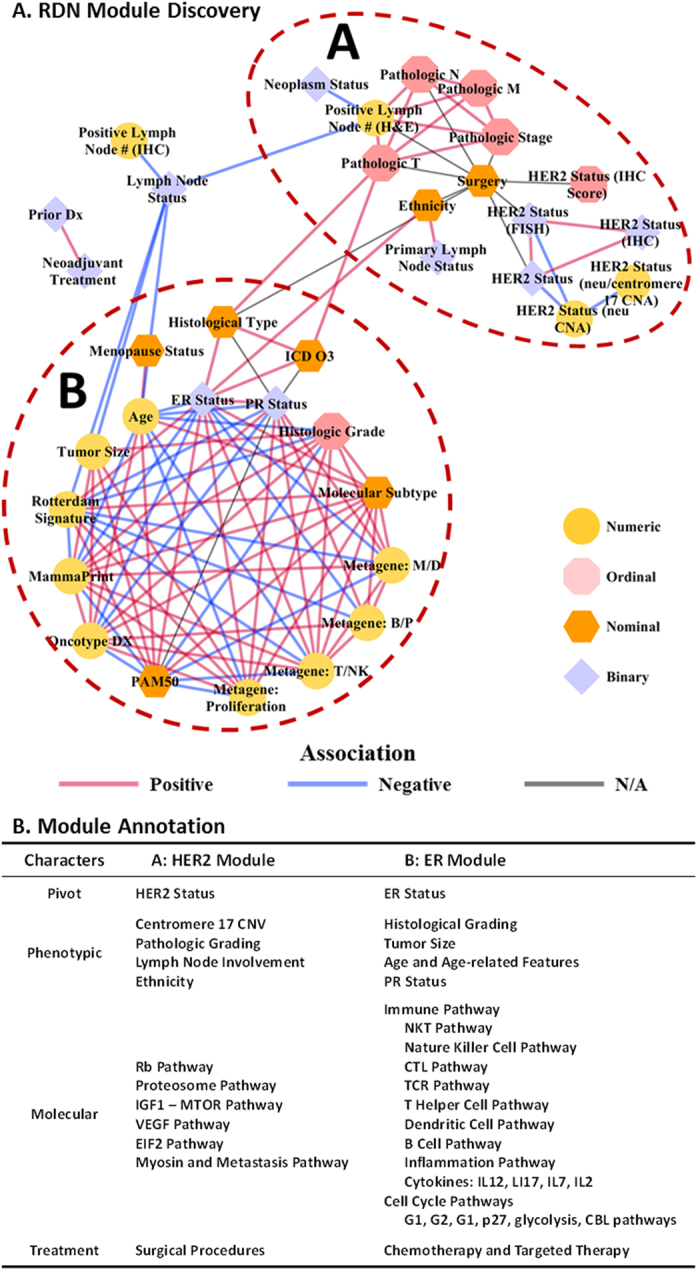
RDN module detection and annotation. (**A**) Visualization of the RDN topology of biomedical concepts of different data types (numeric, ordinal, nominal, and binary, represented in node shapes); association polarity (positive, negative, or not applicable); and the HER2 module (**A**) and ER module (**B**). (**B**) Characters of the HER2 and ER modules: pivot concepts, phenotypic features, molecular underpinnings, and most common treatments.

Strong associations among features were visualized as a relational dependency network (Fig. 5A). The ER module (module A) and the HER2 module (module B) were discovered using the Girvan-Newman’s modularity algorithm (p-value < = 0.0001, Supplement Section 6). The ER module was dominated by ER status, molecular signatures, histologic grading, age and age-related features, and tumor size, with ER status as the “pivot” feature at the center of the module. The HER2 module (module B) was composed of HER2 status, centromere 17 copy number variation, pathologic grading, and lymph node involvement, with HER2 status as the pivot feature.

The signaling pathway annotation enrichment analysis showed that the ER module was associated with a wide range of immune pathways (Fig. 5B), including: tumor surveillance mechanisms (e.g. cytotoxic T cells, natural killer cells, and dendritic cells); responses of tumor tissues (e.g. apoptosis, cell cycle arrest, and inflammation); regulatory mechanisms of tumor-specific immune responses (e.g. T helper cell types 1 and 2, associated T cell apoptosis); complex communications among immune cells through interleukin (IL)17, IL12, IL2, IL7, and IL22; and the involvement of other types of immune cells, including B cells, monocytes, and granulocytes. In contrast, the HER2 module was associated with typical oncogenic phenotypes, such as cell growth (EIF2, IGF1-MTOR, and proteasome pathways); proliferation (Rb pathway), angiogenesis (VEGF pathway); and invasion (myosin and metastasis pathways) (Fig. 5B). Therefore, the two modules described the same patient from two different perspectives – one from the *in situ* immunity patterns, and the other from the tumor growth and invasion patterns.

Decisions regarding surgical procedures were strongly associated with pathologic staging, HER2 status, and histologic types. These features included pathologic stage using the TNM system^36^ and the corresponding pathologic features, including tumor size, lymph node involvement, and metastasis status. In contrast, ER and PR status (as well as histologic grades and molecular signatures, where reported), although critical to patient prognosis, did not significantly affect the decisions of patients and physicians in clinical practice.

These “clinically omitted” features reflect a gap between clinical practice and the current molecular profiling achievement. The explosive growth of omics data and rapid progress in bioinformatics approaches have generated numerous molecular signatures for subtyping patients through molecular-level evidence. Many of these signatures provide important information about mechanistic differences in disease among individuals and have demonstrated strong clinical potential. However, none of these breast cancer molecular signatures and molecular subtyping actually significantly affects clinical decisions regarding surgeries. A major reason could be that most molecular signatures were developed for identifying suitable drugs, not types of surgeries. For example, the Oncotype DX score was established to predict responses to chemotherapy and the risk of distant metastasis.

Our results suggested that even for such a relatively small RDN (44 nodes), the challenges of data sparseness and heterogeneity could be addressed, and biologically and clinically meaningful knowledge could be extracted for further research and validation.

### RDN-based patient subtyping for better therapeutic design

Using the ER module as an example, we further illustrated how modules of heterogeneous features from disparate resources integrative contribute to better therapeutic designs. We first demonstrated that the RDN module detection allowed cross-cohort integration of features whose associations could not be directly measured. Then we showed that such integration was biologically meaningful by gene set enrichment analysis using BioCarta signaling pathway signatures. Finally, we demonstrated the clinical relevance of the detected ER module by using the associated signatures to stratify responses to tamoxifen-related therapies. We chose these therapies as the use case for the ER module because tamoxifen, an ER antagonist, is the typical therapy for patients with ER-positive breast cancer.

**Figure 6:**
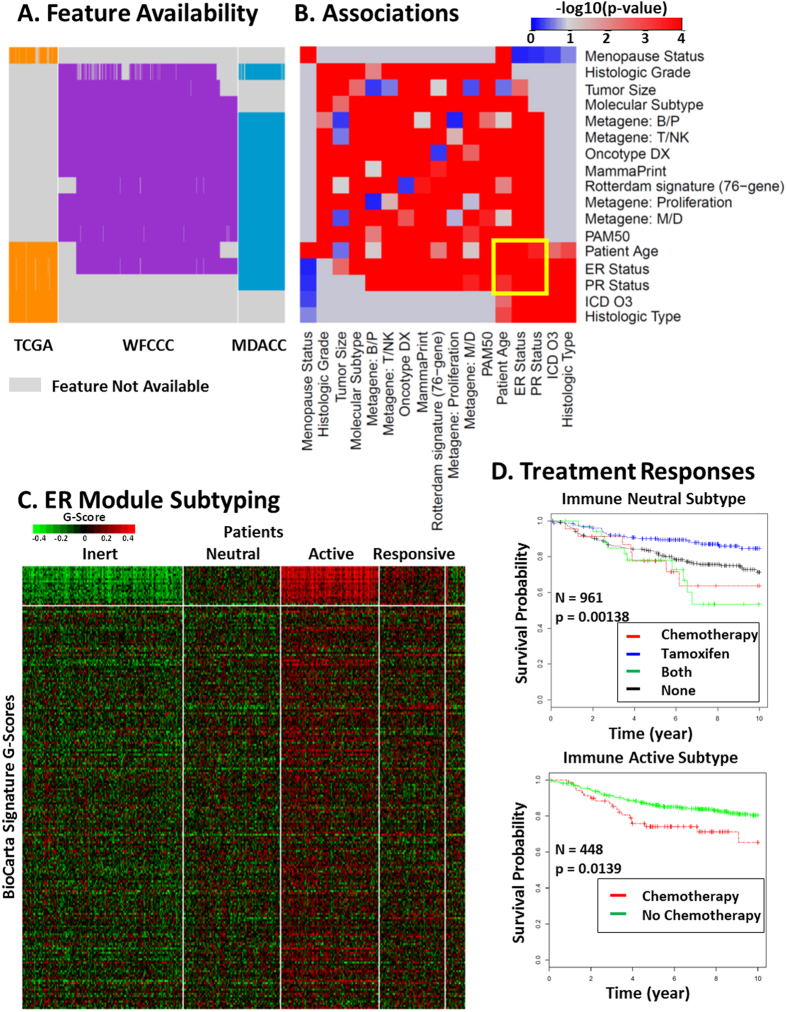
ER module discovery and patient subtyping for drug responses. (**A**) Feature availability among the TCGA (orange), WFCCC (purple), and MDACC (blue) cohorts. Feature names are shown at the right. Unavailable features are labeled in gray. (**B**) Pairwise associations among features in the ER module presented in 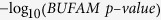. The yellow box highlights common associations shared by two groups of associations. (**C**) Patient subtyping using the BioCarta signatures (top region) associated with the ER module. These signatures revealed four subtypes: Immune Inert, Neutral, Active, and Responsive. (**D**) Differential treatment responses assessed by Kaplan-Meier survival analysis of the WFCCC cohort, using distant-metastasis-free survival time as the index. Survival curves of the Immune Neutral (top) and Active (bottom) Subtypes under different treatments are presented and labeled with patient numbers and log-rank test p-values. More details are given in the Supplement.

We showed that the BUFAM approach could reliably and sensitively discover associations among features by integrating data from diverse and structurally heterogeneous datasets. However, in many cases, due to the lack of data, we could not directly measure associations between two intrinsically related features. This challenge was addressed by projecting these BUFAM-discovered pairwise associations onto a unified RDN, allowing indirect associations among features based on their connections. We used network-related approaches^37^,^38^ such as the Girvan-Newman network community discovering algorithm. As an example, the availability map of the ER module’s features (Fig. 6A) showed that three clinical features (ICD O3 code in the EMR, histologic type, and menopause status) were unique to the TCGA dataset. All molecular signatures (e.g. PAM50 subtypes, metagene signatures, Oncotype DX score, etc.) and some clinical features (e.g. histologic grade and tumor size) were missed in the TCGA dataset. Thus, associations between the TCGA-unique and the TCGA-absent features in the ER module were not measurable by BUFAM (gray region in Fig. 6B).

The RDN module detection allowed indirectly associating such features. The TCGA cohort shares three clinical features with the other two cohorts: patient age, ER status, and PR status (Fig. 6A). These “pivot” features (the 5 × 5 feature region highlighted by the yellow box in Fig. 6B) demonstrated strong associations with both TCGA-unique features (the small red region of 5 × 5 features, at the bottom right corner, assessed using TCGA dataset) and TCGA-absent features (the large red region of 14 × 14 features, according to the WFCCC and MDACC (MD Anderson Cancer Center) datasets). Thus, associations between the TCGA-unique features and the TCGA-absent features were indirectly assessed by the RDN network module detection approach according to the association strength between these features and the shared “pivot” features. Signaling pathway enrichment analysis showed that such indirect associations of the same RDN modules of features were actually driven by common biological mechanisms (i.e., tumor immunity for the ER module) instead of the arbitrary assembling of features.

The clinical relevance of the ER module could not be directly assessed using the associated features because none of the three cohorts we used includes all these features (Fig. 6A). Thus, we used the ER-module-enriched BioCarta signaling pathway signatures (Fig. 5B) to test whether the ER module could help to distinguish differential responses to different treatment strategies. Four subtypes were discovered: the Immune Inert, Neutral, Active, and Responsive subtypes, based on the patient’s immune response (suppressed, neutral, strongly active, and modestly active, respectively; Fig. 6C). Each subtype demonstrated different drug responses to the four types of adjuvant treatments: chemotherapies, tamoxifen, chemotherapies plus tamoxifen), and none (no adjuvant treatment) (Kaplan-Meier plots in Supplement: Figures S7–10). For example, patients with the Immune Neutral subtype showed very diverse responses to different treatment plans. They significantly benefited from tamoxifen treatment (blue curve) but not as much from chemotherapies (red curve for chemotherapy alone, and green curve for tamoxifen + chemotherapy combination) (Fig. 6D). In contrast, patients with the Immune Active subtype did not from tamoxifen treatment (black curve for no treatment, blue curve for tamoxifen) (Supplement Figure S8). Furthermore, chemotherapies (red curves) actually increased the risk of tumor relapse in this subtype (analyzed using Cox’s proportional hazards test) (Supplement Figure S11).

The ER module exhibited strong associations with patient immunity patterns and responses to tamoxifen and chemotherapy, consistent with the literature. Adams and colleagues^39^ reported that ER status had a complex impact on cell functions such as immunity and proliferation. Bates *et al*. reported more regulatory (FOXP3 positive) T cells in ER-negative than ER-positive patients; once the latter group showed high levels of regulatory T cells, they were at higher risk for tumor relapse^40^. Teschendorff and colleagues reported a good prognostic subtype of breast cancer was characterized by ER-negative tumor status and overexpression of genes that confer immunity^41^. Nagalla *et al*.^9^ and Schmidt *et al*.^42^, among others, confirmed the important roles of immunity in cancer relapse, metastasis, and patient survival. Furthermore, ER signaling was found to directly regulate immunity^43^, meaning that ER-targeted hormone therapies might alter the immune pattern in breast cancer patients. For example, tamoxifen has complex effects on immune function, including potentially shifting the immunity pattern from cellular to humoral^44^.

Chemotherapies also modulate the immune system^45^. On one hand, intensive chemotherapy tends to suppress the immune system due to bone marrow toxicity caused by lymphocyte depletion^46^. On the other hand, chemotherapies also directly or indirectly stimulate some immune functions which might contribute to their anti-cancer capacity. For example, Chan *et al*.^47^ and Tsavaris *et al*.^48^ reported that the taxanes paclitaxel and docetaxel boost mixed lymphocyte reactions and effects of natural killer and lymphokine-activated killer cells, while suppressing levels of IL-1 and tumor necrosis factor-α (TNF-α). Therefore, it appears that the ER module is strongly related with immune signatures and these signatures could predict patient drug responses.

Our results also suggest that the conflicting reports regarding ER and immunity- related drug responses might due to heterogeneity in patients. The immune active subtype patients might benefit most from natural immune surveillance, and were very sensitive to the immune suppression effects of chemotherapies. How combination therapy (tamoxifen with chemotherapies) affects patient survival is also not clear. For example, Osborne and colleagues reported antagonism between tamoxifen and melphalan or fluorouracil^49^, and other trials using this combination of treatments found inconsistent results^50^. In contrast, Fisher *et al*.^51^ found that the combinations of tamoxifen and L-phenylalanine mustard or fluorouracil benefited elderly patients with ER-positive or PR-positive tumors. Our results identified for the first time the specific patient sub-population (the Immune Neutral subtype) who responded best to tamoxifen but had a much worse prognosis once chemotherapies were combined with tamoxifen. Thus, patient stratification according to the ER-model related immune signatures demonstrated the high potential of the data discovered from the RDN analysis.

### Comprehensive evaluation of the discovered associations

Reliably evaluating associations among biomedical features in big datasets is challenging, largely due to the lack of reliable ground truth and often inconsistent literature reports. For this exploratory work, we developed a comprehensive strategy to check the discovery from three aspects: 1) Cross-domain validation. We used data from the WFU EMR data, an independent dataset used for clinical purposes, to examine discovered associations. Different from traditional strategies of using similar datasets in the research domain for cross-validation, the EMR dataset was more independent and thus stricter; 2) Translation-oriented validation. We examined the clinical relevance and the translational potentials of the discovered knowledge using inter-cohort cross-validation. In details, we identified patient subgroups on one cohort (TCGA) according to the discovered associations, and then applied the subtyping model to another cohort (WFCCC) to examine whether stratified patients showed different drug responses. This evaluation of effectiveness examined whether the obtained knowledge was clinically actionable. 3) Literature-based validation. We limited validation to those features that had been well studied in the literature. We used literature-based validation for Oncotype DX Score-related associations. However, we did not heavily rely on evidence from literature, since the widely existing inconsistent (and often controversial) reports undermine confidence in such “dry validation”. Our comprehensive validation strategy provided more reliable and practical evaluation of the biologic and clinical values of the BUFAM-based RDN approach as well as the discoveries described herein.

## Conclusions

We developed a bootstrapping-based unified feature association approach and used it to analyze three major breast cancer data repositories. We incorporated demographic, diagnostic, treatment and molecular features of patients, analyzed the associations of these features across heterogeneous datasets, visualized the discovered knowledge as a pan-BRCA (Breast Cancer) relational network, and validated these discoveries with electronic medical record data. Two sets of strongly associated features, the ER and the HER 2 modules, were discovered and annotated with the underpinning molecular signaling signatures. Four novel immunity-related breast cancer subtypes were discovered based on the ER module and demonstrated clinical potential for designing precision therapy schemes. The BUFAM-derived RDN modeling approach demonstrated the ability to detect clinically actionable associations across highly heterogeneous biomedical big data. Our approach for the first time overcomes the obstacle of big-data-associated feature heterogeneity, and allows efficient utilization of diverse datasets for unified knowledge discovery.

## Data and Methods

### Breast cancer data

#### Research datasets

Breast cancer patient data from four repositories were used in this study: TCGA: The Cancer Genome Atlas^10^, RNA microarray dataset, n = 530 patients; RNA-seq data, n = 1,094 patients (see Supplement Section 7); MDACC: MD Anderson Cancer Center^11^,^12^, n = 508 patients; WFCCC: Wake Forest Comprehensive Cancer Center^9^, n = 1954 patients; and WFU EMR of Wake Forest Baptist Medical Center. The EMR-related study was approved by the institutional Ethical Committee (IRB00025669). Written informed consent from all the participants in the study was acquired prior to the collection of samples and medical history, or was waived. Overall, 266 features were selected, merged, mapped, and harmonized into to 46 pan-BRCA key features and stored in the DrugSig Translational Data Warehouse.

#### Electronic medical records

The WakeOne EMR data of breast cancer patients were obtained with appropriate approval from the Institutional Review Board of Wake Forest University.

### BUFAM-derived RDN modeling for feature association discovery

#### Overall pipeline

The overall pipeline of the BUFAM-derived RDN knowledge discovery is illustrated in Figure S2. Briefly, 266 heterogeneous features of breast cancer patients across three biomedical big data repositories were used. Pairwise relations were uniformly assessed using the BUFAM algorithm, validated using the WakeOne EMR data, and used to establish an RDN for module detection and indirect association discovery. The underlying mechanisms of the discovered modules were revealed by gene set enrichment analysis using BioCarta signaling pathway signatures^52^. The enriched pathway signatures of a discovered module (the ER module) were used to stratify patients for drug treatment prediction.

#### BUFAM association algorithm

We developed BUFAM (Fig. 1A and BUFAM p-value, Supplement), an approach for unified pairwise learning of feature relations with bootstrapping. The relationships among features were evaluated in a pairwise fashion using the portion of the data where both features were available. To reflect disparities in data types, we used data-type-specific statistical models to measure specific associations among features. As listed in Fig. 1B, five different correlation measures were used for the 10 distinct combinations of the four data types – the Spearman test, one-way test, Chi-square test, Wilcoxon-Mann-Whitney rank sum test, and linear by linear association test. To allow unified association measurement across these statistical tests, we used a bootstrapping approach (Fig. 1A) to normalize the biases introduced by the uneven sample sizes and the difference among correlation tests. For each feature pair, original data were randomly resampled to generate a negative control distribution. The original correlation test value was represented as the Z-test p-value by comparing with the control distribution. All correlations among features were able to be fairly screened using the corresponding post-bootstrapping p-values. Thus, the BUFAM approach, which uses the data-type-specific statistical tests to address the challenge of data type disparity, and the bootstrapping control to normalize the statistical test disparity, allowed global screening and ranking feature-wise relations.

Formalized mathematical details are provided in Supplement Section 2 (BUFAM p-value).

#### RDN modeling approach

Significant feature-to-feature relations, evaluated by BUFAM p-values, were extracted to establish a relational dependency network (RDN). After adjusting for multiple testing, associations of false discovery rates (FDRs) less than 0.01 were used to build the RDN model. Features were associated as modules using Girvan-Newman’s modularity clustering algorithm^38^. Major statistical features and details of analysis are available in Supplement Section 3 (Statistics of the signed RDN).

#### Validation using EMR dataset

Data were validated using the WakeOne EMR data. A total of 28 associations were validated using corresponding association methods.

#### Comparison with KNN imputation

Numeric features were used to compare the performance of the BUFAM association before and after KNN imputation^17^ using the R impute package (R version 1.42.0, Bioconductor version: Release 3.1)^53^.

#### Comparison with meta-analysis

BUFAM was performed on each separately and all cohorts together. Results were compared based on three aspects of feature associations: coverage, sample size used for each, and statistical significance.

### Biological and clinical relevance

#### Module annotation

Discovered feature modules were annotated using a customized gene set enrichment analysis (GSEA) analysis, with the underlying signaling pathways using 217 BioCarta^52^ signaling pathways collected by MSigDB^54^,^55^. Randomized samples were used as controls for false discoveries. For details, see Supplement Section 4 (Modified gene set enrichment analysis for network module annotation).

#### Survival analysis for drug response

Patients were subtyped according to the discovered ER module using the TCGA cohort, and the drug responses of each subtype were compared using the WFUCCC dataset. Patients from the TCGA cohort were clustered against the ER module using the module-specific BioCarta signaling signatures and a sparse k-means approach. Four patient subtypes were discovered: Immune Inert, Neutral, Active, and Responsive subtypes. WFUCCC patients were classified into these subtypes accordingly, and their responses to four types of adjuvant treatment strategies (chemotherapies, tamoxifen, both, and none) were evaluated by Kaplan-Meier survival analysis with log-rank test and the Cox proportional hazards model, using the distant metastasis-free survival time as the index. More details are provided in Supplement Section 5 (Statistical analysis of Kaplan–Meier survival curves).

### Availability of resources

All data except the WakeOne EMR were available using SQL through the DrugSig Translational Data Warehouse (PostgreSQL) hosted by Center for Bioinformatics & Systems Biology, Wake Forest School of Medicine. For more details, see our website (http://ctsb.is.wfubmc.edu/express/db/drugsig.htm).

## Additional Information

**How to cite this article**: Chen, H. *et al*. Relational Network for Knowledge Discovery through Heterogeneous Biomedical and Clinical Features. *Sci. Rep.* **6**, 29915; doi: 10.1038/srep29915 (2016).

## Supplementary Material

Supplementary Information
