# Supplementary material for: Relational Network for Knowledge Discovery through Heterogeneous Biomedical and Clinical Features
Source: Sci Rep. 2016 Jul 18;6:29915. doi: 10.1038/srep29915 (PMC4947904; doi:10.1038/srep29915)
Supplement: Supplementary Information [file srep29915-s1.pdf]

Relational Network for Knowledge Discovery through Heterogeneous Biomedical and Clinical Features

Huaidong Chen[1 §], Wei Chen[2 §], Chenglin Liu[2], Le Zhang[1], Jing Su[2]* and Xiaobo Zhou[2]*

* Corresponding Authors: Jing Su: Jing.Su.66@gmail.com; Xiaobo Zhou: xizhou@wakehealth.edu

[1] School of Computer and Information Science, Southwest University, Chongqing, 400715, China,

[2] Center for Bioinformatics & Systems Biology, Division of Radiological Sciences, Wake Forest University School of Medicine, Winston-Salem, NC, 27127, USA

[§] Equal contribution.

Table of Contents

1. Formalization of the heterogeneous association problem. 3
2. BUFAM p-value. 5
3. Statistics of the signed RDN. 7
4. Modified gene set enrichment analysis for network module annotation. 8
5. Statistical analysis of Kaplan–Meier survival curves. 11
6. Randomization test for Girvan-Newman’s modularity. 12
7. Case Study: Comparison of BUFAM and Meta-analysis. 13

Figure S1: Unified biomedical feature association analysis on heterogeneous big data. . 14

Figure S2: Flowchart of BUFAM-based RDN modeling 15

Figure S3: Heterogeneous and sparse features across the three clinical biomedical big datasets. 16

Figure S4: The WFU EMR validation of association between the ER status and the ethnicity. 17

Figure S5: The WFU EMR validation of association between the clinical procedures and the ethnicity. (p-value <= , χ^2 test) 18

Figure S6: Balance analysis for signed graph. 19

Table S1. KEGG Module Annotation. 20

Table S1. Reactome Module Annotation. 21

Figure S7: The ER Kaplan-Meier survival analysis for the drug responses of the Immune Inert subtype patients. 22

Figure S8: The ER Kaplan-Meier survival analysis for the drug responses of the Immune Neutral subtype patients. 23

Figure S9: The ER Kaplan-Meier survival analysis for the drug responses of the Immune Active subtype patients. 24

Figure S10: The ER Kaplan-Meier survival analysis for the drug responses of the Immune Responsive subtype patients. 25

Figure S11: Cox's proportional hazards regression analysis. 26

Figure S12: Randomization control for the discovered modules using Girvan-Newman approach. 27

Figure S13: Case Study: BUFAM vs Meta-analysis. 28

References 29

1. Formalization of the heterogeneous association problem.

As visualized in Figure S1, given a group of features $F = \{f_A, f_B, f_C, f_D, f_E \cdots\}$, the true associations among these features, represented as a set of signs $R = \left\{r_{XY} = \{1, -1\} | f_X, f_y \in F\right\}$, form a signed graph $G = \{F, R\}$, with features as vertices and associations as edges (the graph at bottom-right). The data types of these features can be numeric, binary, ordinal, or nominal.

To identify the relations among these features, a series of independent sampling efforts are carried on and N datasets, denoted as $D_1, D_2, \cdots, D_N$, are generated. Each dataset $D_l \equiv \{(s_i, f_X) | s_i \in S_l, f_X \in F_l\}$ covers a specific subset of features $F_l \subseteq F$ measured from a set of samples $S_l \equiv \{s_i\}$. Thus, the overall super dataset $D\{S, F\} \equiv \bigcup_{l=1}^{N} D_l = \{(s_i, f_X) | s_i \in S, f_X \in \mathrm{F}\}$ where the set of all samples $S \equiv \bigcup_{l=1}^{N} S_l$. Datasets usually share some features but not samples. That is, $\exists\ F_l, F_k \subseteq F$ that $F_l \cap F_k \neq \emptyset$ but $\forall\ S_l, S_k \subseteq S$, $S_l \cap S_k = \emptyset$.

The <u>problem</u> is to determine the association set R according to the super dataset $D\{S, F\}$. Theoretically, the <u>goal</u> is to maximize the similarity $Sim(G, \hat{G})$ between the true set of associations G and the estimated $\hat{G} = \{\hat{F}, \hat{R}\}$:

$$\underset{\hat{G} = \{\hat{F}, \hat{R}\}}{\operatorname{argmax}} \operatorname{Sim}(G, \hat{G})$$

In reality, due to lack of reliable ground truth, we used the similarity between discovered associations on independent datasets as a proximate and empirical measure of the performance of the developed BUFAM-based RDN approach as well as the reliability of the discovered associations.

The similarity was measured by the modified Jaccard similarity coefficient between the estimated and the true associations:

$$J(R_1, R_2) \equiv \frac{|R_1 \cap R_2|}{|E(R_1) \cup E(R_2)|} \quad (1)$$

where $|\cdot|$ stands for the size of a set (the amount of elements in the set), and $E(R) \equiv \{e_{XY} | r_{XY} \in R\}$ denotes a set of unsigned edges of the corresponding signed edges in R. Spectral analysis based similarity metrics[1,2], which may provide marginal gain of measure accuracy, were not used in this work due to the tremendous computational cost as a result of the exponential complexity of such approaches.

The three challenges due to data heterogeneity are:

1) Heterogeneous association measurements due to the heterogeneous data types. That is, if two different statistical scores $J_{A,B}$ and $K_{C,D}$ are used to evaluate the association strength $r_{A,B}$ and $r_{C,D}$ with the statistical power P_J and P_K, how to define a normalization function $T(\cdot)$ so that $P_{T(J)} = P_{T(K)}$.
2) Heterogeneous statistical power due to heterogeneous data availability across features. That is, the normalization function $T(\cdot)$ should be insensitive to sample sizes.
3) Identify associations between features that do not share samples due to heterogeneous data availability. That is, for an existing association r_{XY} between a pair of features f_X and f_Y, if $\forall\, D_l \subseteq D$, $\{f_X, f_Y\} \not\subset F_l$, how to identify r_{XY}.

2. BUFAM p-value.

The BUFAM p-value is defined as the exact p-value of the corresponding permutation test of a specific statistical score. Let

$$S = (s_1, s_2, \cdots s_n)$$

be the full set of all samples. Assume f and g are the two features of interest. $S_{f,g}$, the largest subset of S that shares both features,

satisfies

$$\forall\, s_i \in S_{f,g}, f(s_i) \neq \text{NA and } \; g(s_i) \neq \text{NA}$$

and

$$\forall\, s_j \in S \text{ but } s_j \notin S_{f,g}, \text{ either } f(s_i) = \text{NA or } g(s_i) = \text{NA}$$

where $f(s_i)$ and $g(s_i)$ are the values of features f and g of sample s_i, respectively. An NA value indicates the value of the corresponding feature for the specific sample is missing. Let $r_{\boldsymbol{f},\boldsymbol{g}}$ be a statistic score describing the association strength between features f and g on $S_{f,g}$:

$$r_{\boldsymbol{f},\boldsymbol{g}} \equiv \text{cor}(\boldsymbol{f}, \boldsymbol{g})$$

where $\text{cor}(\cdot\,,\ \cdot)$ generally represents an association measure (five measures were used in this work, including Spearman test[3], one-way test[4], Chi-square test[5], Wilcoxon Mann Whitney rank sum test[6], and linear by linear association test[7] for corresponding data types as delineated in Figure 1 B), and the two vectors $\boldsymbol{f}$ and $\boldsymbol{g}$ represents the corresponding values of the features f and g for samples in $S_{f,g}$, that is,

$$\boldsymbol{f} = \{f(s_i) | s_i \in S_{f,g}\}$$

and

$$\boldsymbol{g} = \{g(s_i) | s_i \in S_{f,g}\}.$$

The negative control distribution for a specific association between two features were generated by bootstrapping. In detail, let

$$\widehat{\boldsymbol{F}} = \left(\widehat{\boldsymbol{f}}^{(\mathbf{1})}, \widehat{\boldsymbol{f}}^{(\mathbf{2})}, \cdots \widehat{\boldsymbol{f}}^{(\boldsymbol{m})}\right)$$

be the negative control dataset of feature f generated by resampling of $\boldsymbol{f}$ for samples in $S_{f,g}$ for m times. The corresponding statistical scores of the negative controls are

$$\hat{R}_{\boldsymbol{f},\boldsymbol{g}} \equiv \left\{r_{\widehat{\boldsymbol{f}}^{(\boldsymbol{k})},\boldsymbol{g}} | \widehat{\boldsymbol{f}}^{(\boldsymbol{k})} \in \widehat{\boldsymbol{F}}\right\}.$$

The null hypothesis is that features f and g are independent, therefore the original statistic score $r_{\boldsymbol{f},\boldsymbol{g}}$ and that of the negative control $r_{\widehat{\boldsymbol{f}}^{(\boldsymbol{k})},\boldsymbol{g}}$ (in which the data of feature f are randomly permuted) follow the same distribution; the alternative hypothesis is that features f and g are associated therefore $r_{\boldsymbol{f},\boldsymbol{g}}$ and the $r_{\widehat{\boldsymbol{f}}^{(\boldsymbol{k})},\boldsymbol{g}}$ follow different distributions. The BUFAM p-value is defined as the two-tailed permutation p-value:

$$\hat{p}_{f,g} \equiv \min\left(\sum_{r_{\widehat{\boldsymbol{f}}^{(\boldsymbol{k})},\boldsymbol{g}} \in \hat{R}_{\boldsymbol{f},\boldsymbol{g}}} \mathbf{1}_{R_u}\left(r_{\widehat{\boldsymbol{f}}^{(\boldsymbol{k})},\boldsymbol{g}}\right), \sum_{r_{\widehat{\boldsymbol{f}}^{(\boldsymbol{k})},\boldsymbol{g}} \in \hat{R}_{\boldsymbol{f},\boldsymbol{g}}} \mathbf{1}_{R_l}\left(r_{\widehat{\boldsymbol{f}}^{(\boldsymbol{k})},\boldsymbol{g}}\right)\right) \Big/ m$$

where

$$R_u = \left\{r_{\widehat{\boldsymbol{f}}^{(\boldsymbol{k})},\boldsymbol{g}} | r_{\widehat{\boldsymbol{f}}^{(\boldsymbol{k})},\boldsymbol{g}} \in \hat{R}_{\boldsymbol{f},\boldsymbol{g}} \text{ and } r_{\widehat{\boldsymbol{f}}^{(\boldsymbol{k})},\boldsymbol{g}} \geq r_{\boldsymbol{f},\boldsymbol{g}}\right\}$$

and

$$R_l = \left\{r_{\widehat{\boldsymbol{f}}^{(\boldsymbol{k})},\boldsymbol{g}} | r_{\widehat{\boldsymbol{f}}^{(\boldsymbol{k})},\boldsymbol{g}} \in \hat{R}_{\boldsymbol{f},\boldsymbol{g}} \text{ and } r_{\widehat{\boldsymbol{f}}^{(\boldsymbol{k})},\boldsymbol{g}} \leq r_{\boldsymbol{f},\boldsymbol{g}}\right\}$$

are the subsets of $\hat{R}_{\boldsymbol{f},\boldsymbol{g}}$ whose elements are no less or no greater than $r_{\boldsymbol{f},\boldsymbol{g}}$, respectively, and

$$\mathbf{1}_A(x) \equiv \begin{cases} 1 & \text{if } x \in A \\ 0 & \text{if } x \notin A \end{cases}$$

is the indicator function for $A \subseteq X\{x\}$.

3. Statistics of the signed RDN.

Overall, there were 110 edges in the RDN, 45 of them represented positive associations (red), 24 negative (blue), and 41 not applicable (gray, mainly involving nominal features).

Balance analysis for signed graph. A signed graph is called balanced if every circle in the graph has an even number of negative signs, otherwise it is called “frustrated”. Since the full analysis of balance (or, the degree of frustration) is an NP-hard (non-deterministic polynomial-time hard) problem, and most large signed graphs reflecting real-word associations were not fully balanced, we instead focused on the smallest circle (i.e., triangles) to detect immediate inconsistence. It is known that, as a simplified case of Harary’s Theorem[8], a complete signed graph is balanced if and only if all embedded triangles are balanced. Therefore, screening triangles for frustration cases allows detection of the immediate (i.e., with smallest circle) and thus most significant imbalanced cases. As shown in Figure S6, among the 186 triangles, 32 had 3 positive edges, 59 had 1 positive and 2 negative edges, 47 had 1 positive and 2 N/A edges, 35 had 1 negative and 2 N/A edges, and 14 had 3 N/A edges. All determined triangles (that is, without N/A edges) were found balanced. This result showed that the BUFAM approach was highly consistent across heterogeneous datasets and data types.

4. Modified gene set enrichment analysis for network module annotation.

We customized the gene set enrichment analysis algorithms defined in Subramanian et. al. 2005 [9] and Lamb et. al. 2006 [10] by robustly including gene expression levels.

Data preparation. The log2 transformed microarray-based gene expression data for each cohort were patient-wisely quantile normalized, and then by gene-wisely normalized using Z-statistics. Normalization of patient-wise quantile normalization neutralized the impact of outliers (i.e., genes of some patients might show extremely high or low expression levels comparing with the rest). The gene-wise Z-statistics removed the intrinsic difference and variance of absolute gene expression levels among genes by centering the expression levels of each gene of a cohort at 0 and rescaling the variance to 1. For genes that were measured by multiple probes, the average values were used. Such two-step normalization was important for incorporating the gene expression levels into the gene set enrichment analysis.

Gene set enrichment analysis. Given the normalized gene expression data of descending order $X = \{x_1 \geq x_2 \geq \cdots \geq x_N\}$ of a sample (patient) and the gene list of a signature $S = \{g_1, g_2, \cdots, g_M\}$, the empirical cumulative distribution functions of the expression levels of genes *in* and *not in* the signature S were defined as

$$P_{hit}(S,i) = \frac{\sum_{\substack{g_j \in S \\ j \leq i}} |x_j|}{\sum_{g_j \in S} |x_j|}$$

and

$$P_{miss}(S,i) = \frac{\sum_{\substack{g_j \notin S \\ j \leq i}} |x_j|}{\sum_{g_j \notin S} |x_j|}$$

respectively. The gene set enrichment score ES was defined as the largest difference between the two empirical cumulative distributions P_{hit} and P_{miss}. Let the i_{max}'th gene be the one in the descending gene expression list X where the largest difference was observed, that is,

$$\max\left(\text{abs}(P_{hit} - P_{miss})\right) = \text{abs}(P_{hit}(S, i_{max}) - P_{miss}(S, i_{max})),$$

we define

$$ES \equiv P_{hit}(S, i_{max}) - P_{miss}(S, i_{max}).$$

The false discovery rate of the enrichment score between a patient and a signature was defined as the estimated exact p-value using the Monte Carlo negative controls generated by randomizing gene labels of the gene expression data 1,000 times, similar to the definition of the BUFAM p-value.

Network module annotation. Each RDN module was annotated by the BioCarta signaling pathway signatures using a BUFAM-like approach. The goal was to identify BioCarta signaling signatures that were enriched in a RDN module. The null hypothesis was that a BioCarta signaling signature is not associated with a module, so the associations between the GSEA enrichment score (ES) of this signature and the features in the module follow the same distribution with those calculated from the resampled values. The alternative hypothesis was that the signature is associated with a significant amount of features in the module. Therefore, we first calculate the two-tailed permutation p-values for pairwise associations between module-related features and the 217 BioCarta signatures using Spearman associations for all samples across the three datasets that shared both features signatures. We used 1000-time permutation to analyze values of the feature and the original values of the signature ESs from the same samples as the negative control group,

as described in BUFAM algorithm. The p-values of each signature were summarized as the overall module-to-signature p-value. Signatures of permutation p-values ≤ 0.05 were used to annotate the module.

KEGG[11,12] and Reactome[13,14] signaling pathway signatures were also used for comparison. As shown in Table S1 and Table S2, databases for each pathway showed distinct focuses, while the general biological meanings were consistent. Both databases support the concept that the ER module demonstrated strong associations with immune effects, checkpoint controls, and hormone- and immune-modulated cell growth events, while the HER2 module showed typical cell proliferation mediated by growth factor receptors. Protein degradation and oxidation functions (BioCarta: Proteasome; KEGG: Lysosome, Peroxisome; Reactome: Protein Oxidations) were also major characteristics of the HER2 module. The KEGG database provided more metabolic details, while Reactome signatures were either more general (such as “Immune System” and “Cytokine Reactions”) or more molecule-specific (such as “DAG” and “IP3”).

5. Statistical analysis of Kaplan–Meier survival curves.

With respect to Figures S7 through S10, we applied log-rank tests and Cox proportional hazard tests to checking the significance of associations among the four treatment strategies and the immune subtypes. P-values of log-rank tests for Figure S7, S8, and S9 were 0.106, 0.001, and 0.108, respectively. Thus, different treatment mechanisms can generate an evident survival diversity corresponding to the immune neutral subtype. In addition, Cox hazard ratios and related p-values between 3 immune subtypes (immune positive, immune negative, and immune neutral) and 4 treatment strategies (chemotherapy, tamoxifen, both, and no treatment) are calculated in Figure S11. Under the condition of no treatment, breast cancer patients of the immune-positive subtype had greater probability of survival, while those of the immune-negative subtype had a lower probability of survival.

6. Randomization test for Girvan-Newman's modularity.

To test if the two modules identified using Girvan-Newman approach were statistically significant, we used randomization to generate a negative control. We randomized the node labels across the two modules so that the generated two modules had the same numbers of nodes (15 and 17 nodes, respectively) for 10,000 times and calculated the modularity of each randomized control. The distribution of the modularity of the randomized control was shown in Figure S12. The position of the modularity of the Girvan-Newman approach (0.363) at the distribution was indicated by the red arrow. The exact p-value of the discovered modules is less than 0.0001.

Modularity[15] is defined as:

$$Q = \frac{1}{2m}\sum_{vw}\left[A_{vw} - \frac{k_v k_w}{2m}\right]\delta(c_v, c_w) = \sum_{i=1}^{c}(e_{ii} - a_i^2)$$

where A is the adjacent matrix of a graph of n nodes, m edges, and c modules (communities), v and w are two nodes, k_v and k_w are their degrees, and c_v and c_w are the modules they belong to, $c_v, c_w \in [1, c]$, and $i \in [1, c]$ is the ith module. The δ-function is defined as:

$$\delta(c_v, c_w) = \begin{cases} 1, \text{if } v \text{ and } w \text{ belong to the same module} \\ 0, \text{if } not \end{cases}$$

The fraction of edges that connect to nodes in community i is defined as:

$$a_i = \frac{1}{2m}\sum_{v} k_v \delta(c_v, i)$$

and the fraction of edges that connect community i and j is defined as:

$$e_{ij} = \frac{1}{2m}\sum_{vw} A_{vw}\delta(c_v, i)\delta(c_w, j)$$

7. Case Study: Comparison of BUFAM and Meta-analysis.

A case, association between Metagene:Proliferation and Age , was further analyzed to demonstrate how associations could be discovered by BUFAM but not by traditional meta-analysis. Associations were test using BUFAM on the whole cohort, and WFUCCC and MDACC cohorts. A new dataset, TCGA RNA-seq (1,094 patients), was used as external validation. Results (Figure S13) showed clear association in BUFAM (p <= 2.2e-16) and WFUCCC (p <= 0.001) cohorts but not in MDACC (p <= 0.11) cohort. The TCGA RNA-seq dataset strongly support the discovered association (p <= 0.0003). That is, traditional meta-analysis was not capable to identify this association (MDACC p <= 0.11) due to smaller sample sizes and thus limited power. In contrast, BUFAM demonstrated strong statistical power (p <= 2.2e-16) and was able to identify this association.

Figure S1: Unified biomedical feature association analysis on heterogeneous big data.

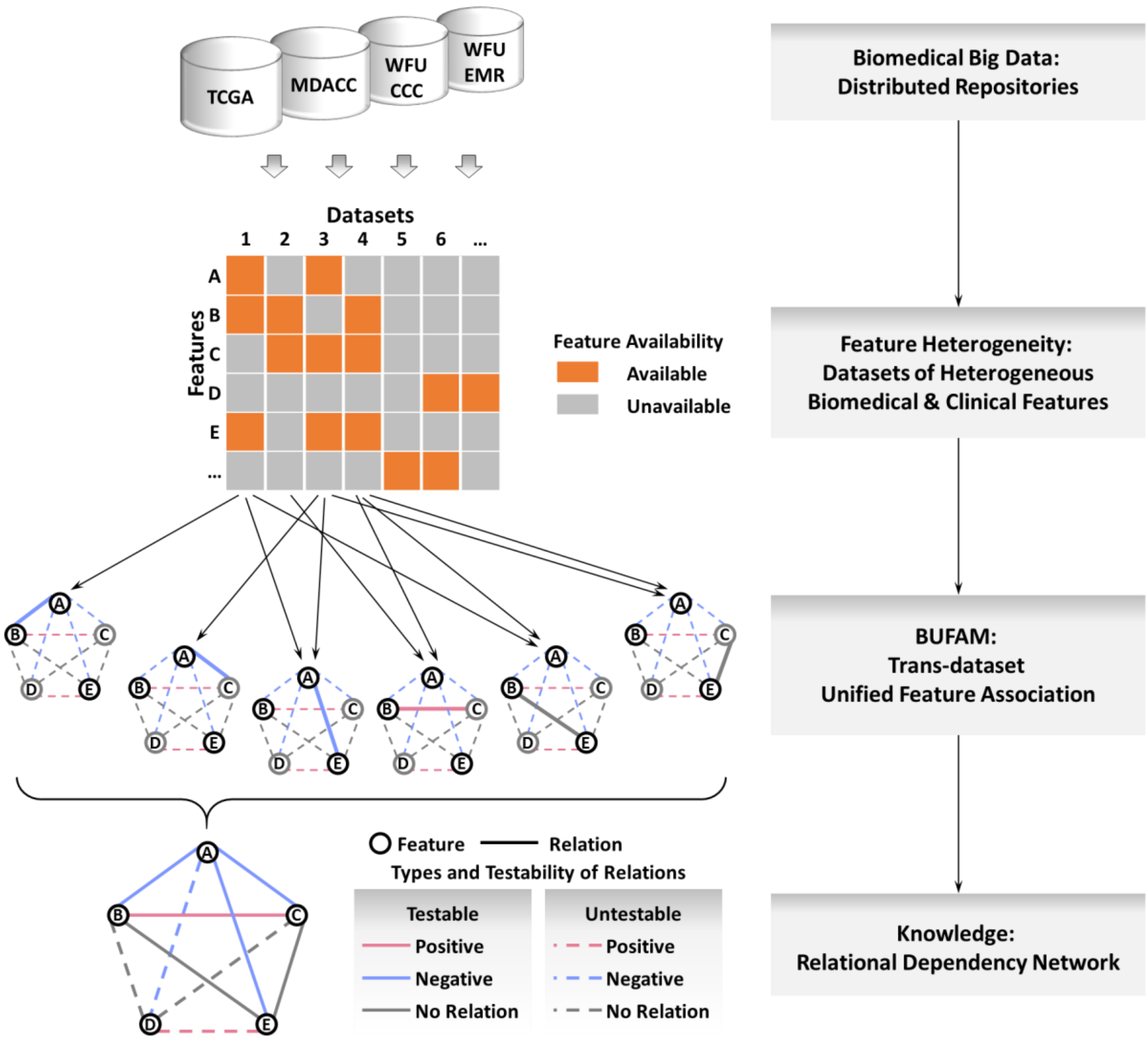

Figure S2: Flowchart of BUFAM-based RDN modeling

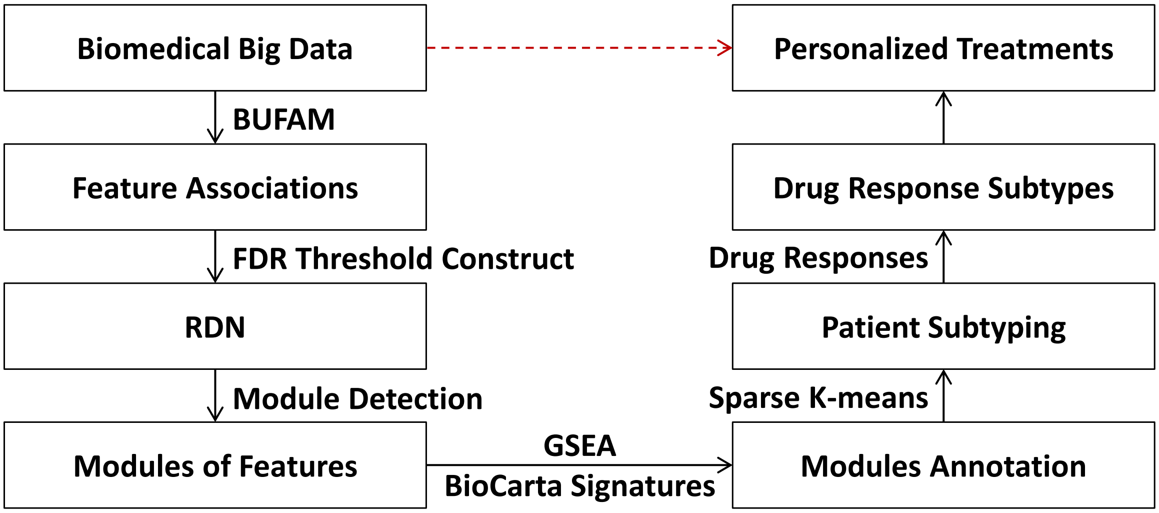

Figure S3: Heterogeneous and sparse features across the three clinical biomedical big datasets.

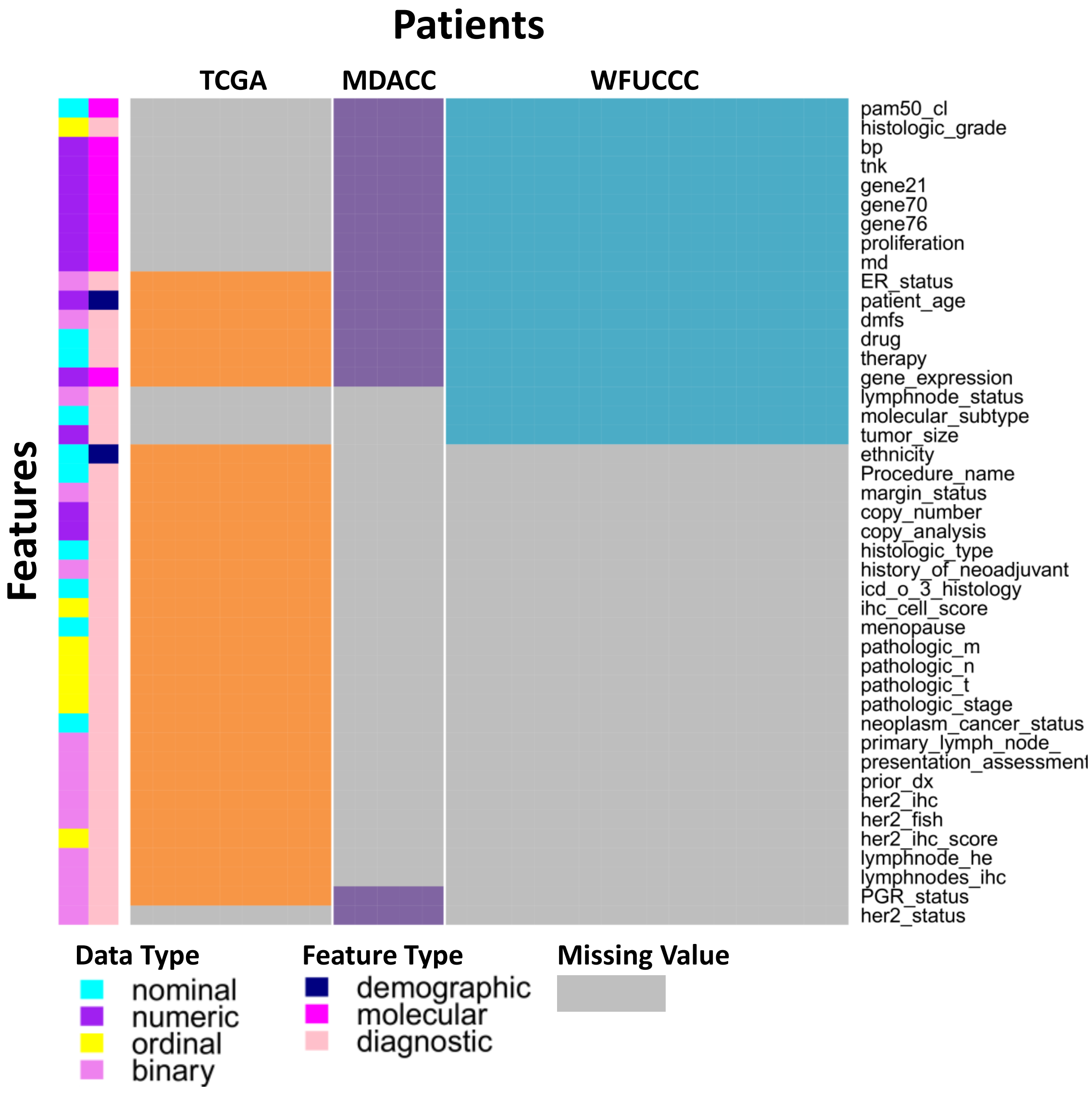

Figure S4: The WFU EMR validation of association between the ER status and the ethnicity.

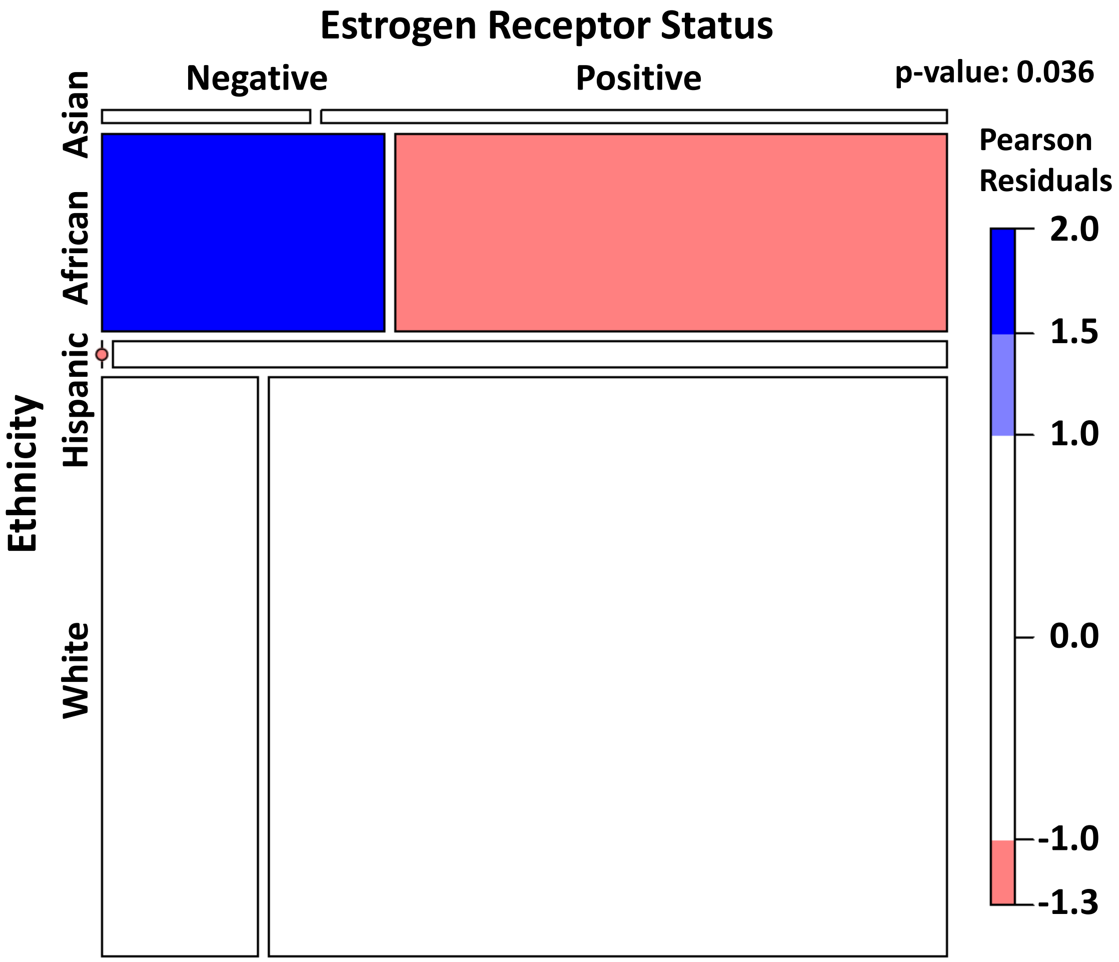

Figure S5: The WFU EMR validation of association between the clinical procedures and the ethnicity. (p-value <= , χ^2 test)

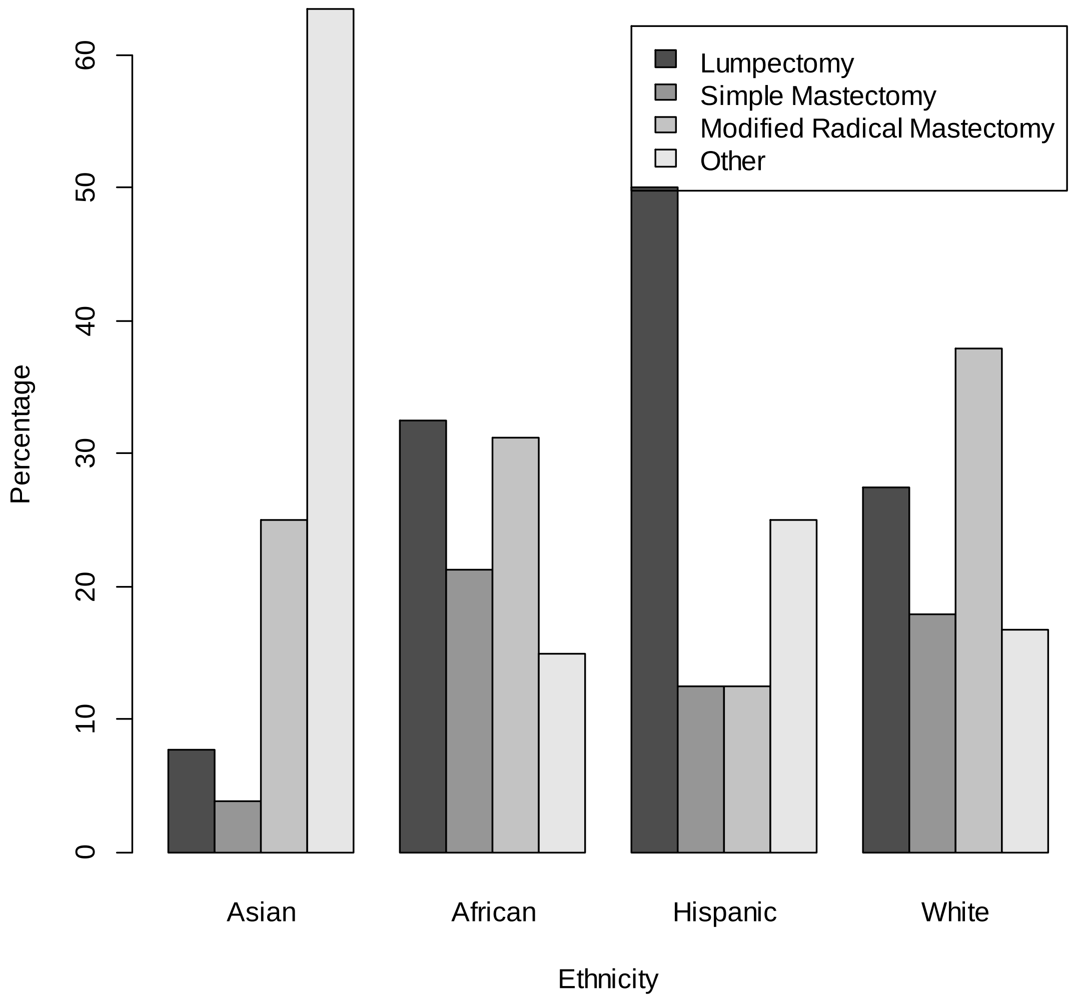

Figure S6: Balance analysis for signed graph.

Cases of embedded triangles were summarized per cases. Among the embedded 186 triangles, 32 were with 3 positive edges, 59 with 1 positive and 2 negative edges, 47 with 1 positive and 2 N/A edges, 35 with 1 negative and 2 N/A edges, and 14 with 3 N/A edges. All determined triangles (that is, without N/A edges) were found balanced.

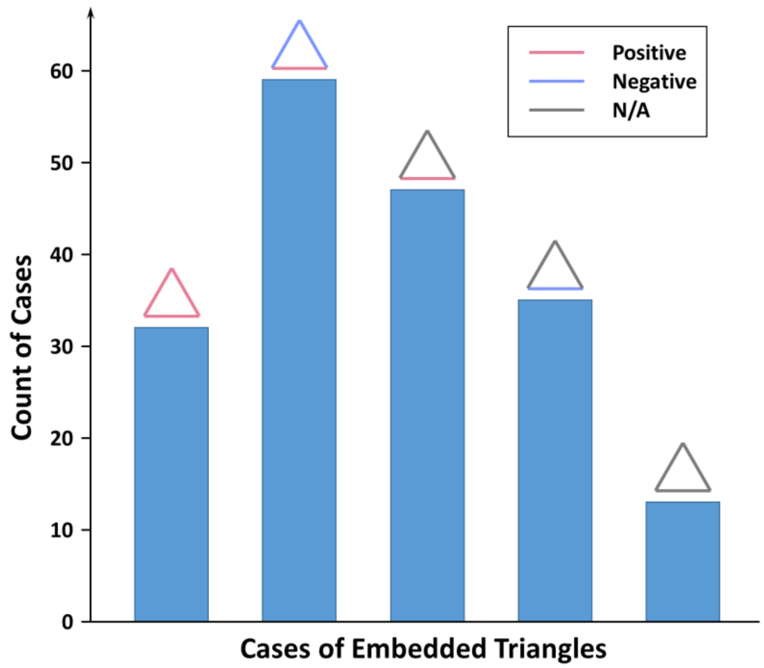

Table S1. KEGG Module Annotation.

KEGG Module Annotation

A: HER2 Module	B: ER Module
	Immune Pathway
	B Cell Receptor Signaling
	Chemokine Signaling
Protein Processing	Cytokine Signaling
Aminoacyl tRNA Biosynthesis	Infection-related Epithelial Signaling
Lysosome, peroxisome	Cell Growth
Adhesion	Amino Acid Biosynthesis
Vascular functions	Cell Cycle
Metabolism Pathway	DNA Replication
Alpha Linolenic Acid, Butanoate,	Fatty Acid Biosynthesis
Glutathione, Nitrogene,	Glycolysis
Phenylalanine, Porphyrin &	GNRH Signaling
Chlorophyll, Retinol	MAPK Pathway
PPAR Signaling	Protein Biosynthesis
Type I & II Diabetes	TGFβ Pathway
Cytokine Signaling	Phosphatidylinositol Signaling
Adipocytokine	Metabolism Pathway
Cytokine/Receptor	Galactose, Glycolipids,
Apoptosis	Glycosaminoglycan,
	PyruvateSelenoamino Acid, Tryptophan
	& Hypotaurine

Table S1. Reactome Module Annotation.

Reactome Module Annotation

A: HER2 Module	B: ER Module
Cell Cycle Pathways<br>Androgen, Creb, DNA Biosynthesis, E2F, EGFR, EGFR/SHC1, ERBB4/SHC1, FGF/FGFR, IGFR, Insulin, p38, Smad 2/3/4 Heterotrimer<br>Oxidations<br>Diabetes Pathways<br>Cytokine: IL6<br>Metabolism<br>Fatty Acyl CoA, Lipoprotein, Pyruvate & Citric Acid TCA Cycle	Immune Pathway<br>B Cell Receptor<br>Complement Cascades<br>Cytokine Signaling in Immune System<br>Immune System<br>Interferon (α, β, and γ) Signaling and Regulation<br>Innate Immune<br>TLR Activation<br>Virus Infection<br>Cytokines:<br>Cell Growth<br>Amino Acid & Protein Biosynthesis<br>AMPK, BMP, Notch, PI3K-Akt<br>ATP Formation<br>CDC6 and S/M Phase Coordination<br>Cell Cycle Checkpoints, Cyclin E/G1-S Transition, G0/Early G1, P53/G1<br>DNA Replication<br>Peptide Elongation<br>RNA Biosynthesis and Processing, POL I/II/III Functions and Switching<br>Telomere Maintenance<br>Tubulin<br>Signaling: AMPK, BMP, DAG & IP3, ERKs, Glycoprotein Hormones, G Protein Signaling, Growth Hormone Receptors, Hormone Ligand/Receptor, IKK/NFκB, Notch1, PI3K, RAF/MAPK, SHC, TGFβ<br>Metabolism<br>Carbohydrates, Glucogenesis, Glycolysis, Lipids and Lipoproteins, Sphingolipid, Triglyceride<br>Ion Channels: Calcium, Potassium

Figure S7: The ER Kaplan-Meier survival analysis for the drug responses of the Immune Inert subtype patients.

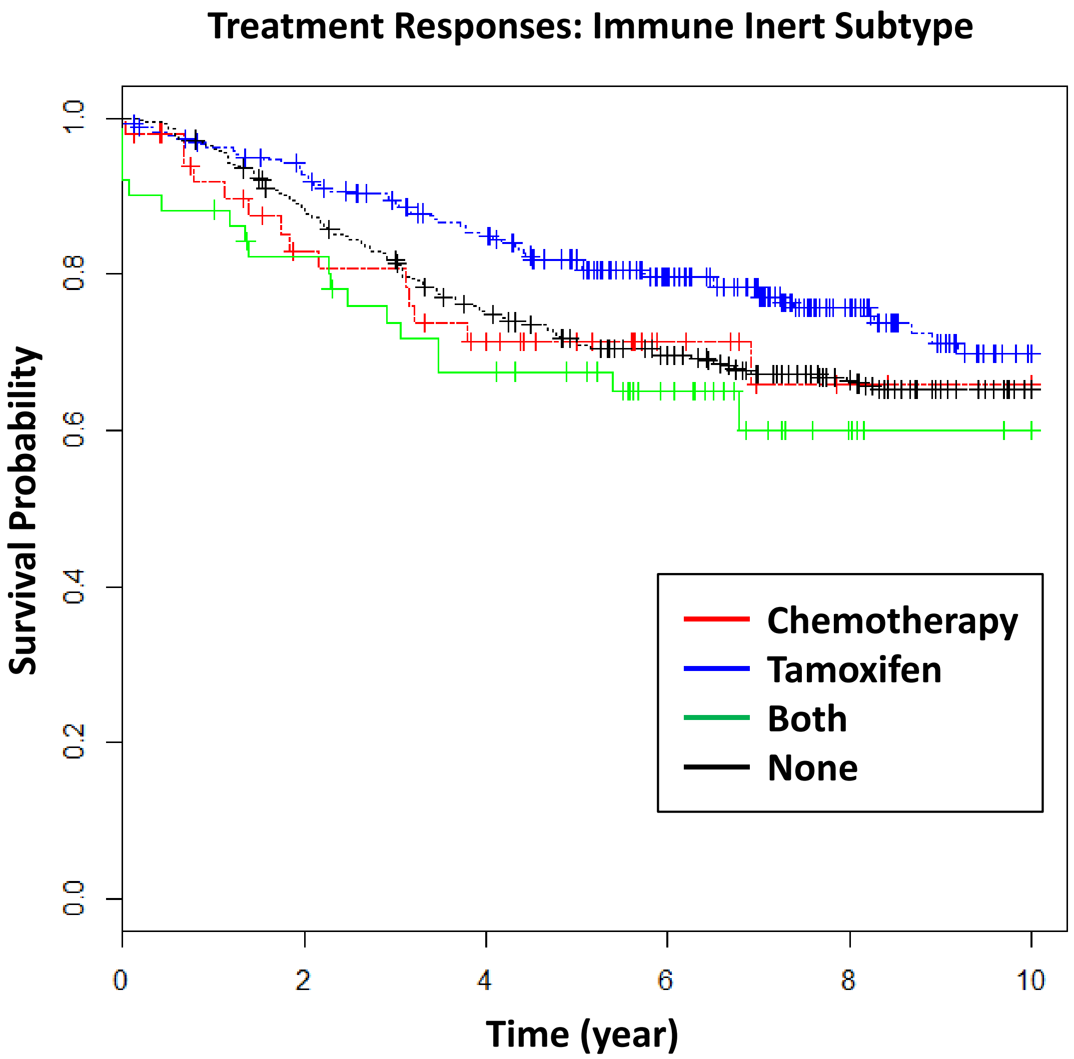

Figure S8: The ER Kaplan-Meier survival analysis for the drug responses of the Immune Neutral subtype patients.

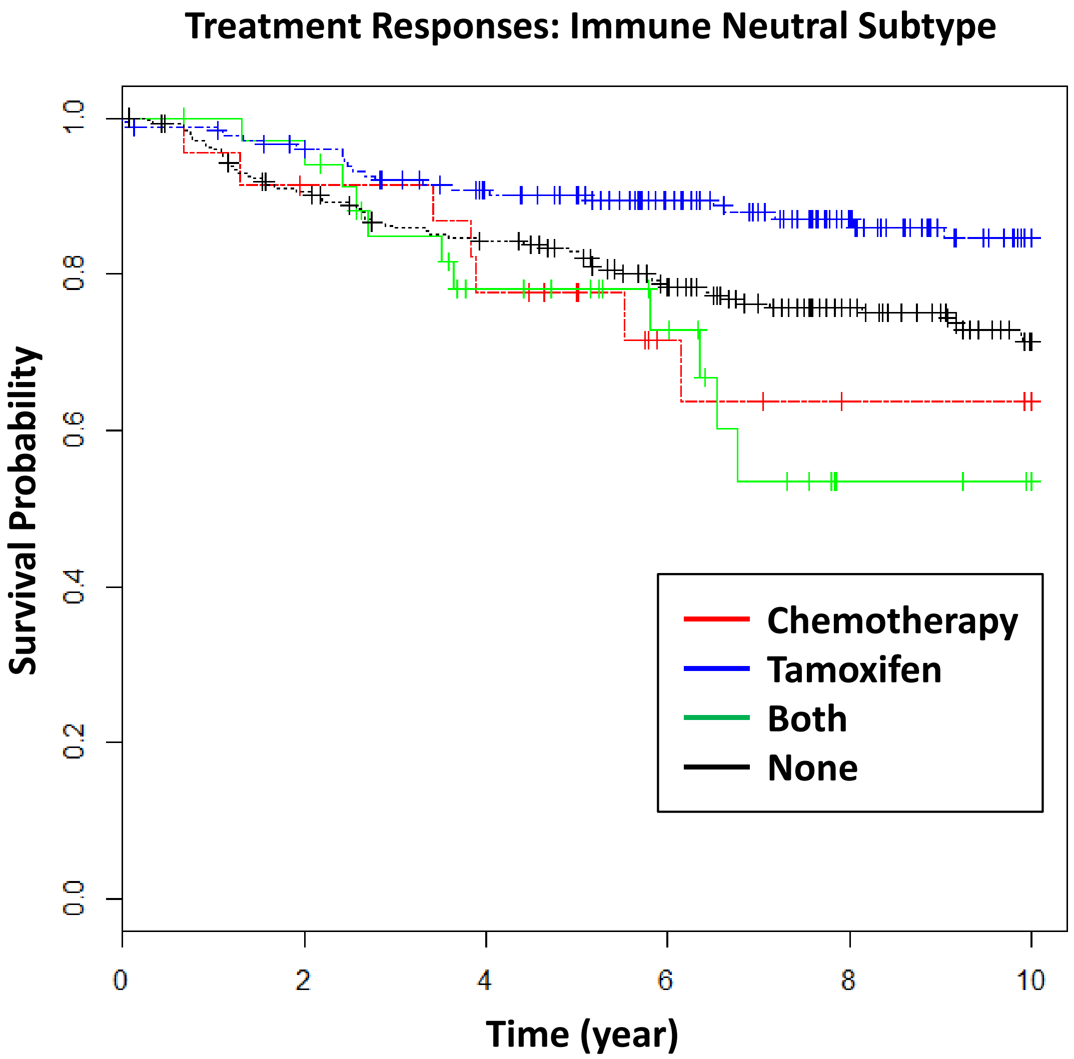

Figure S9: The ER Kaplan-Meier survival analysis for the drug responses of the Immune Active subtype patients.

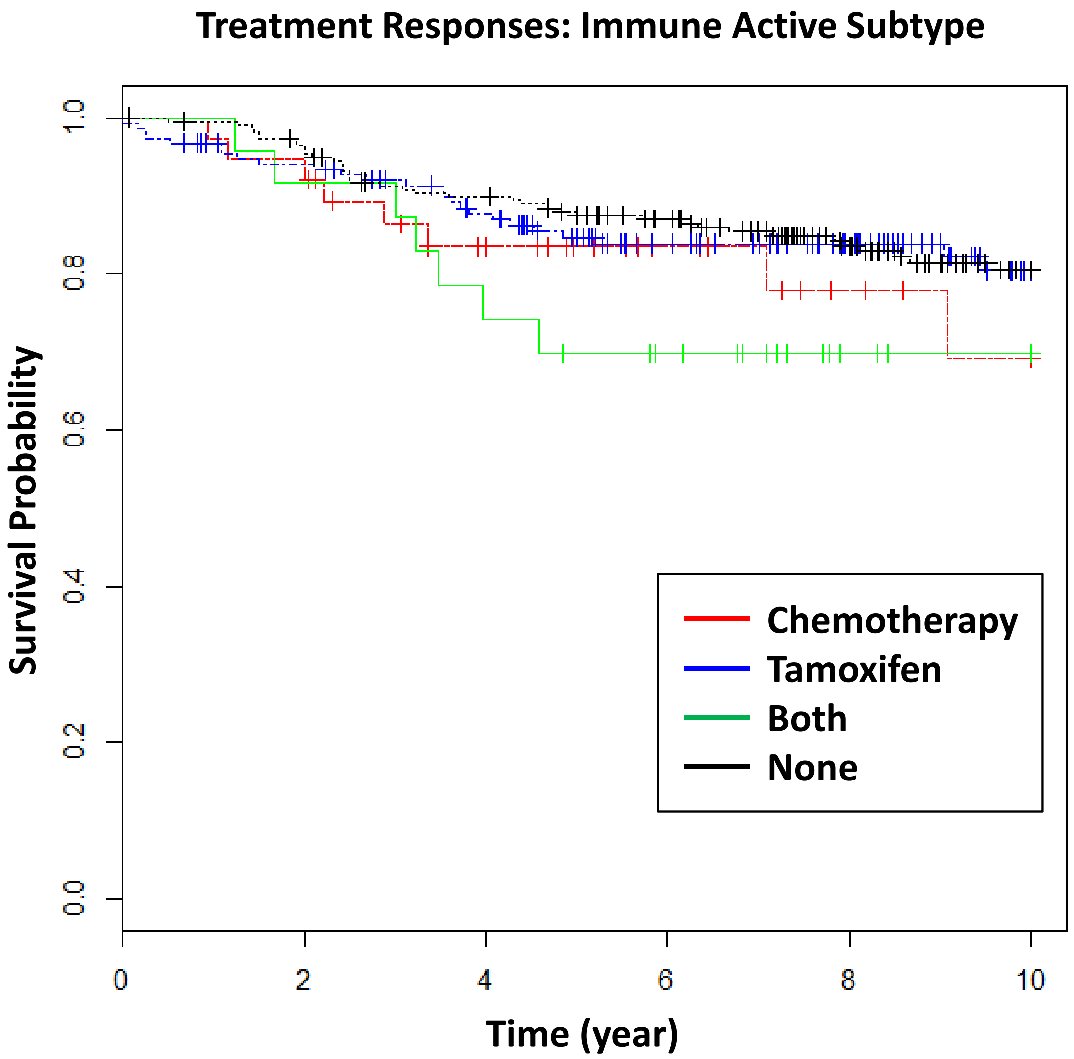

Figure S10: The ER Kaplan-Meier survival analysis for the drug responses of the Immune Responsive subtype patients.

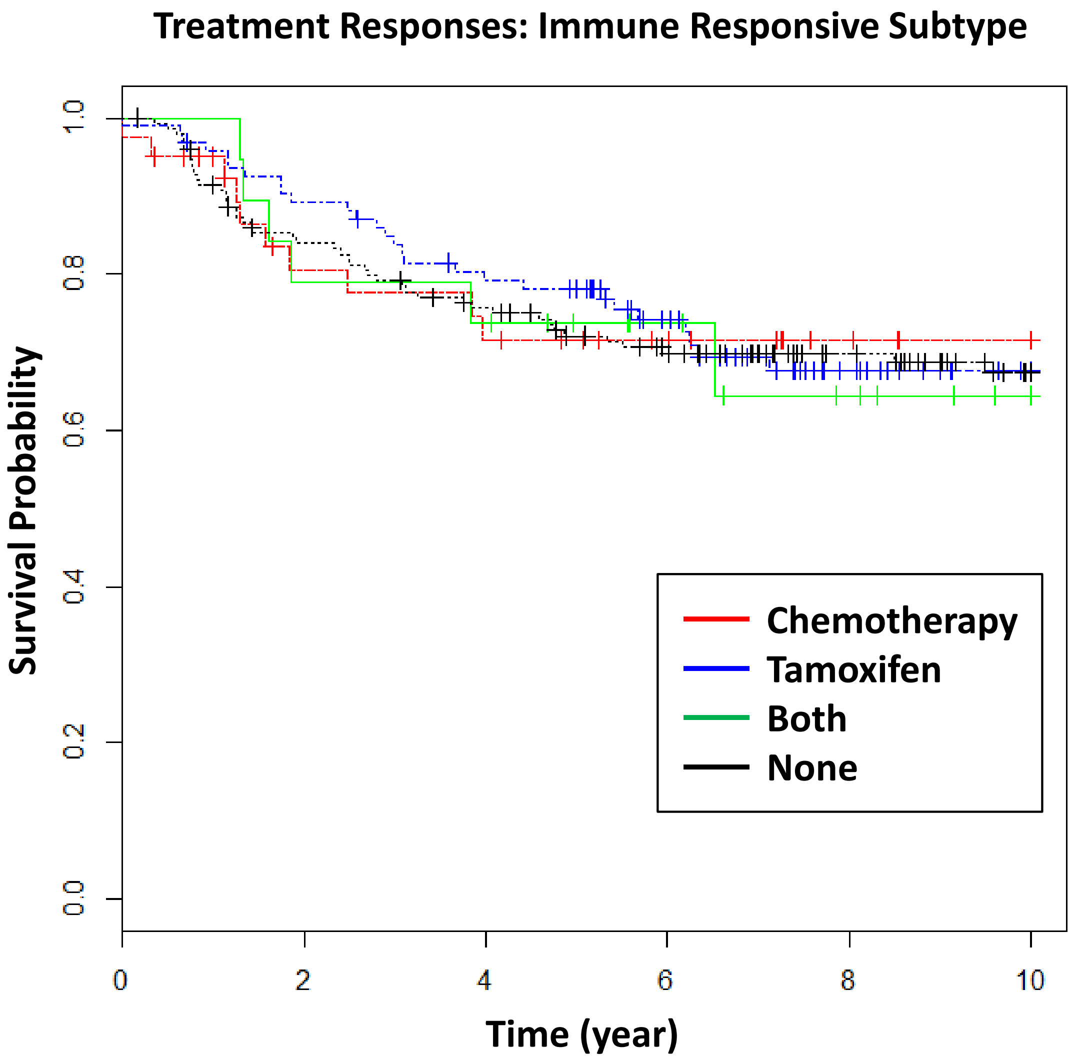

Figure S11: Cox's proportional hazards regression analysis.

Cox’s proportional hazard ratios (beta values, left side) and p-values (right side) for 3 immune subtypes (Immune Positive, Immune negative and Immune Neutral) and 4 treatment strategies (Chemotherapy, Tamoxifen, both Chemotherapy and tamoxifen and no treatment) were demonstrated.

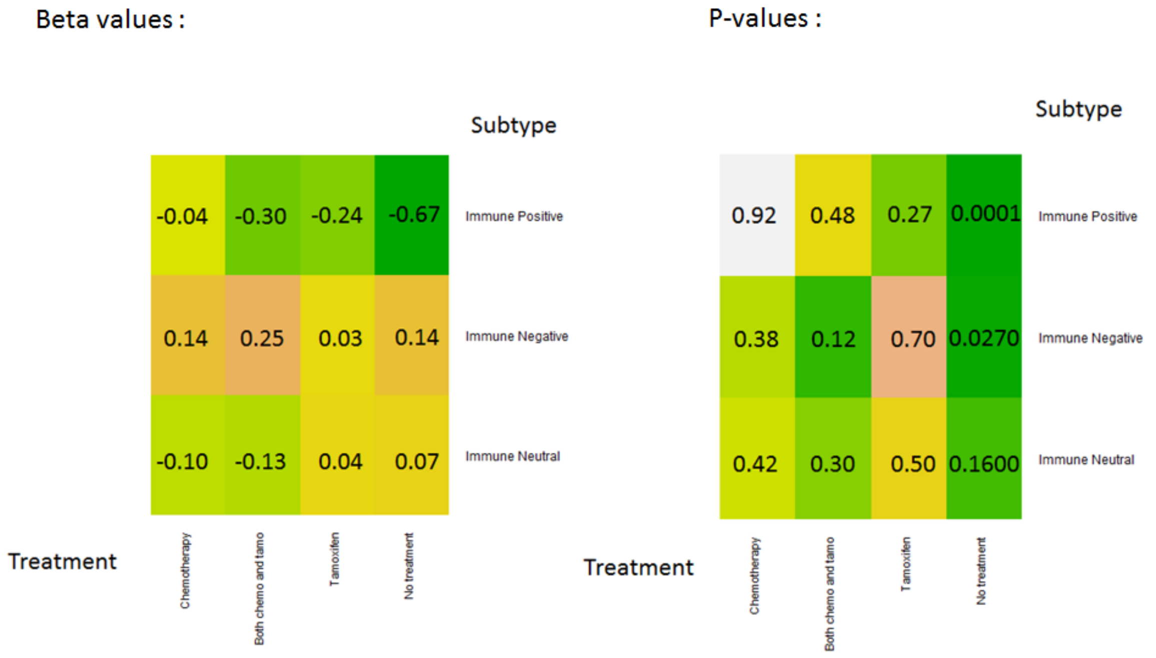

Figure S12: Randomization control for the discovered modules using Girvan-Newman approach.

The distribution of the modularity scores of 10,000 randomization of node labels of the RDN were presented in the histogram. The position of the modularity of the Girvan-Newman approach (0.363) at the distribution was indicated by the red arrow.

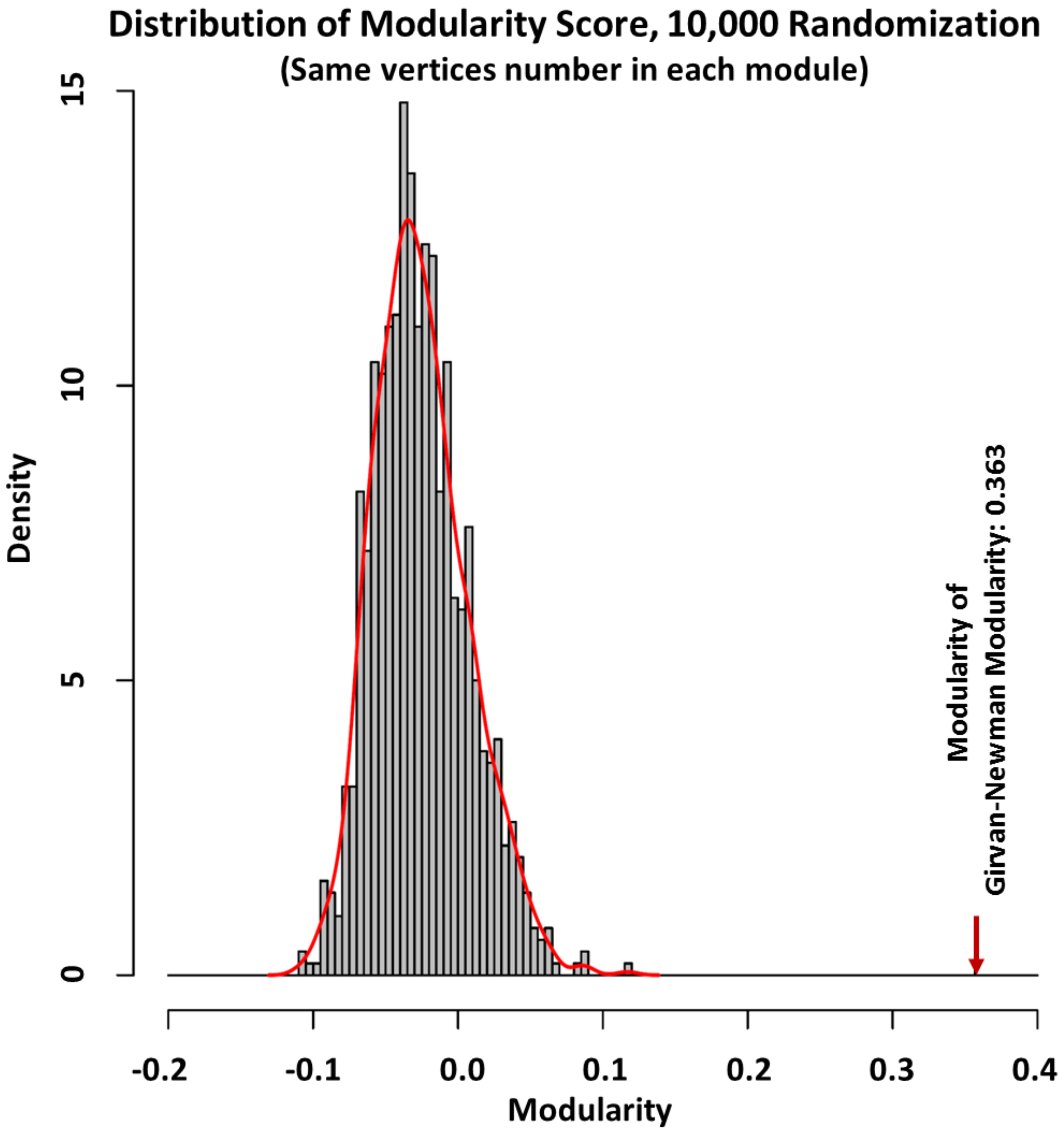

Figure S13: Case Study: BUFAM vs Meta-analysis.

The association between Metagene:Proliferation and Age were analyzed using BUFAM and using WFUCCC and MDACC cohorts, and tested using TCGA RNA-seq data. Circle: median. Error bar: 95% CI.

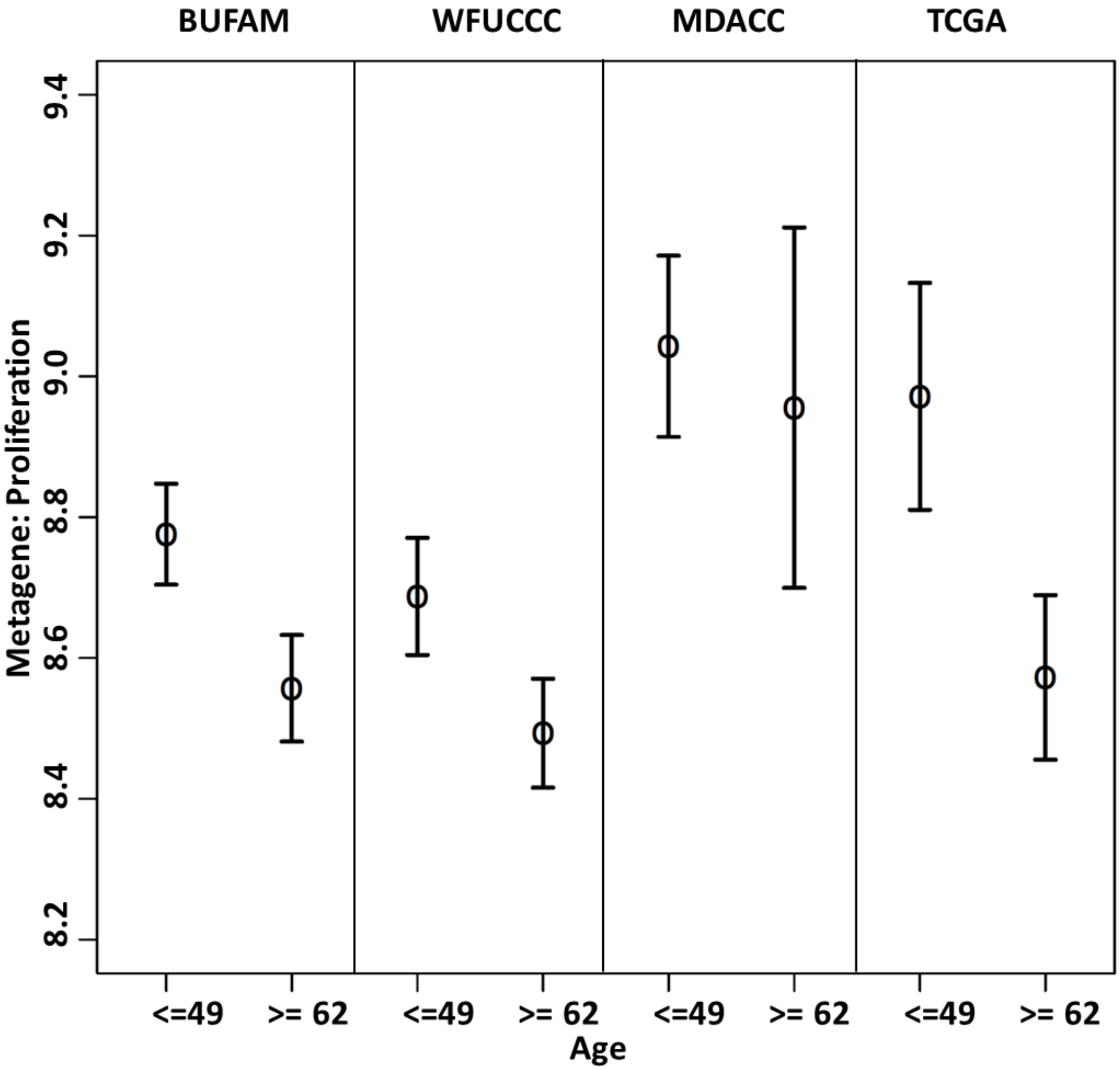

References

1 Kunegis, J. *et al.* 559-559 (SIAM).
2 Blondel, V. D., Gajardo, A., Heymans, M., Senellart, P. & Van Dooren, P. A measure of similarity between graph vertices: Applications to synonym extraction and web searching. *Siam Review* **46**, 647-666, doi:10.1137/S0036144502415960 (2004).
3 Spearman, C. "General Intelligence," Objectively Determined and Measured. *The American Journal of Psychology* **15**, 201-292, doi:10.2307/1412107 (1904).
4 Welch, B. L. ON THE COMPARISON OF SEVERAL MEAN VALUES: AN ALTERNATIVE APPROACH. *Biometrika* **38**, 330-336 (1951).
5 Pearson, K. X. On the criterion that a given system of deviations from the probable in the case of a correlated system of variables is such that it can be reasonably supposed to have arisen from random sampling. *Philosophical Magazine Series 5* **50**, 157-175, doi:10.1080/14786440009463897 (1900).
6 Mann, H. B. & Whitney, D. R. On a test of whether one of two random variables is stochastically larger than the other. *The annals of mathematical statistics*, 50-60 (1947).
7 Agresti, A. & Kateri, M. *Categorical data analysis*. (Springer, 2011).
8 Cartwright, D. & Harary, F. Structural balance: a generalization of Heider's theory. *Psychological review* **63**, 277 (1956).
9 Subramanian, A. *et al.* Gene set enrichment analysis: a knowledge-based approach for interpreting genome-wide expression profiles. *Proceedings of the National Academy of Sciences of the United States of America* **102**, 15545-15550, doi:10.1073/pnas.0506580102 (2005).
10 Lamb, J. *et al.* The Connectivity Map: using gene-expression signatures to connect small molecules, genes, and disease. *Science* **313**, 1929-1935, doi:10.1126/science.1132939 (2006).
11 Kanehisa, M. & Goto, S. KEGG: kyoto encyclopedia of genes and genomes. *Nucleic acids research* **28**, 27-30 (2000).
12 Du, J. *et al.* KEGG-PATH: Kyoto encyclopedia of genes and genomes-based pathway analysis using a path analysis model. *Molecular bioSystems* **10**, 2441-2447 (2014).
13 Joshi-Tope, G. *et al.* Reactome: a knowledgebase of biological pathways. *Nucleic acids research* **33**, D428-432 (2005).
14 Fabregat, A. *et al.* The Reactome pathway Knowledgebase. *Nucleic acids research* **44**, D481-487 (2016).
15 Newman, M. E. J. & Girvan, M. Finding and evaluating community structure in networks. *Phys Rev E* **69**, doi:Artn 026113
Doi 10.1103/Physreve.69.026113 (2004).
